# Innovative applications of advanced nanomaterials in cerebrovascular imaging

**DOI:** 10.3389/fbioe.2024.1456704

**Published:** 2025-01-22

**Authors:** Li Na, Xiaofu Song, Ping Luo, Jingqi Su, Zhicheng Yao

**Affiliations:** ^1^ Department of Neurology, Liaoning Provincial People’s Hospital, Shenyang, China; ^2^ Liaoning Provincial People’s Hospital, China Medical University, Shenyang, China

**Keywords:** nanomaterials, cerebrovascular imaging, metal nanoparticles, magnetic nanoparticles, quantum dots, carbon-based nanomaterials, polymer nanoparticles

## Abstract

Cerebrovascular imaging is essential for the diagnosis, treatment, and prognosis of cerebrovascular disease, including stroke, aneurysms, and vascular malformations. Conventional imaging techniques such as MRI, CT, DSA and ultrasound have their own strengths and limitations, particularly in terms of resolution, contrast and safety. Recent advances in nanotechnology offer new opportunities for improved cerebrovascular imaging. Nanomaterials, including metallic nanoparticles, magnetic nanoparticles, quantum dots, carbon-based nanomaterials, and polymer nanoparticles, show great potential due to their unique physical, chemical, and biological properties. This review summarizes recent advances in advanced nanomaterials for cerebrovascular imaging and their applications in various imaging techniques, and discusses challenges and future research directions. The aim is to provide valuable insights for researchers to facilitate the development and clinical application of these innovative nanomaterials in cerebrovascular imaging.

## 1 Introduction

Cerebrovascular imaging is crucial for diagnosing, treating, and predicting the course of cerebrovascular diseases (Haller et al., 2018; Hage et al., 2016). Conditions like stroke, aneurysms, and vascular malformations often result in significant morbidity and disability, impacting both patients and society. High-resolution, high-contrast imaging is essential for accurate diagnosis and effective treatment. Current techniques include Magnetic Resonance Imaging (MRI) (Sleight et al., 2021), Computed Tomography (CT) (Abbas and Kumar, 2018), Digital Subtraction Angiography (DSA) (Shaban et al., 2022), and ultrasound (Yan et al., 2019). Each has its advantages and disadvantages. CT is fast and offers high resolution, but it has poor soft tissue contrast and carries radiation risks (Ghekiere et al., 2017). MRI provides excellent soft tissue contrast, but scans take longer and it is sensitive to metal implants (Sammet, 2016). DSA is often considered the gold standard, providing detailed images, but it is invasive, complex, and involves radiation exposure (Orru’ et al., 2020). The limitations of these techniques, particularly in resolution, contrast, and safety, motivate researchers to explore and develop more advanced imaging methods for improved diagnosis and treatment of cerebrovascular diseases.

Nanomaterials are materials with at least one dimension in the nanoscale range, approximately 1–100 nm. Due to their minuscule size, they exhibit unique and enhanced properties compared to their bulk counterparts, including increased surface area, quantum effects, and size-dependent optical, electrical, and magnetic behaviors. These characteristics make nanomaterials highly versatile and applicable across various fields. Nanotechnology’s rapid advancement has created new possibilities in biomedicine (Fakruddin et al., 2012; Campos et al., 2020; Sindhwani and Chan, 2021). Nanomaterials, with their unique physical, chemical, and biological properties, show great promise for bioimaging (Liang et al., 2020; Li et al., 2023; Shen et al., 2022). Their small size, large surface area, diverse surface modification options, and excellent biocompatibility offer advantages over traditional materials. In cerebrovascular imaging, nanomaterials have introduced exciting innovations. For example, metallic nanoparticles, with their strong optical and electrical properties, are ideal contrast agents for optical and electronic imaging (Anderson et al., 2019; Padmanabhan et al., 2016; Srinoi et al., 2018). Magnetic nanoparticles, due to their magnetic behavior, enhance MRI contrast (Jeon et al., 2021; Lin et al., 2020; Zhao Z. et al., 2022). Quantum dots, with tunable spectra and high brightness, are promising for fluorescence imaging (Zhao B. et al., 2022; Wang et al., 2022; Chung et al., 2021). Carbon-based nanomaterials like graphene and carbon nanotubes offer excellent electrical conductivity and mechanical strength, making them suitable for multimodal imaging (Srivastava et al., 2021; Gollavelli et al., 2022). Polymer nanoparticles, with their biocompatibility and degradability, are valuable for molecular imaging and targeted therapy (Ong et al., 2021; Haupt et al., 2020; Yang et al., 2020). These nanomaterials enhance imaging resolution, contrast, and specificity through various mechanisms.

This review systematically analyzes recent advances in nanomaterials for cerebrovascular imaging. It explores their applications across different imaging techniques, evaluating their advantages and disadvantages. It also proposes future research directions. The review focuses on several key nanomaterials (Table 1): metallic nanoparticles, magnetic nanoparticles, quantum dots, carbon-based nanomaterials, and polymer nanoparticles. Their applications in MRI, CT, fluorescence imaging, and multimodal imaging are discussed in detail. Challenges such as biocompatibility, targeting, preparation processes, and ethical considerations are also addressed. This review aims to provide valuable insights for researchers and promote further development and application of advanced nanomaterials in cerebrovascular imaging.

**TABLE 1:** Representative nanomaterials.

Nanomaterials	Characteristics	Applications	Advantages	Limitations
Metal nanoparticles	Surface Plasmon resonance and optical properties (Kumar et al., 2018)	Cancer treatment, biological imaging, chemical sensing, and drug delivery (Altammar, 2023)	Strong plasma absorption, Biological system imaging, Determine chemical information on metallic nanoscale substrate (Li et al., 2007)	Difficulty in synthesis Particles instability Impurity (Granqvist and Buhrman, 1976)
Magnetic nanoparticles	Superparamagnetic, high magnetic susceptibility, high coercivity, non-toxicity, biocompatibility, low Curie temperature (Yadollahpour and Rashidi, 2015)	Targeted drug delivery, magnetic hyperthermia, contrast agent for magnetic resonance imaging (Yadollahpour and Rashidi, 2015)	Non-toxicity, Biocompatibility, high-level aggregation in the desired tissue, high effective surface areas and lower sedimentation rates	Tendency to aggregate or the poor magnetization, Certain Degree of toxicity (Cardoso et al., 2018)
Quantum dots	Exceptional photochemical and photophysical properties (Reshma and Mohanan, 2019)	Medical diagnostics, drug delivery (Reshma and Mohanan, 2019)	Desirable photon emission properties, advantageous absorption properties (Reshma and Mohanan, 2019)	Unproductive due to antibody binding, Heavy metals present in the core may be toxic to host (Reshma and Mohanan, 2019)
Carbon-based nanoparticles	Electrical conductivity, high strength, structure, electron affinity, and adaptability (Altammar, 2023)	Tissue engineering, drug delivery, imaging, and biosensors (Multifunctional Carbon)	Unique structural dimensions and excellent mechanical, electrical, thermal, optical and chemical properties (Patel et al., 2019)	The toxicity remains a debated issue (Zhang et al., 2014)
Polymer nanoparticles	Biocompatibility, biodegradability (Zielińska et al., 2020)	Molecular imaging, drug delivery, and targeting cancer research (Lu et al., 2011)	Structures can be modified, with intricate definition over their compositions, structures and properties (Elsabahy and Wooley, 2012)	Stability Issues Toxicity Concerns Complex Manufacturing

## 2 Classification and characteristics of advanced nanomaterials

### 2.1 Metal nanoparticles

**FIGURE 1:**
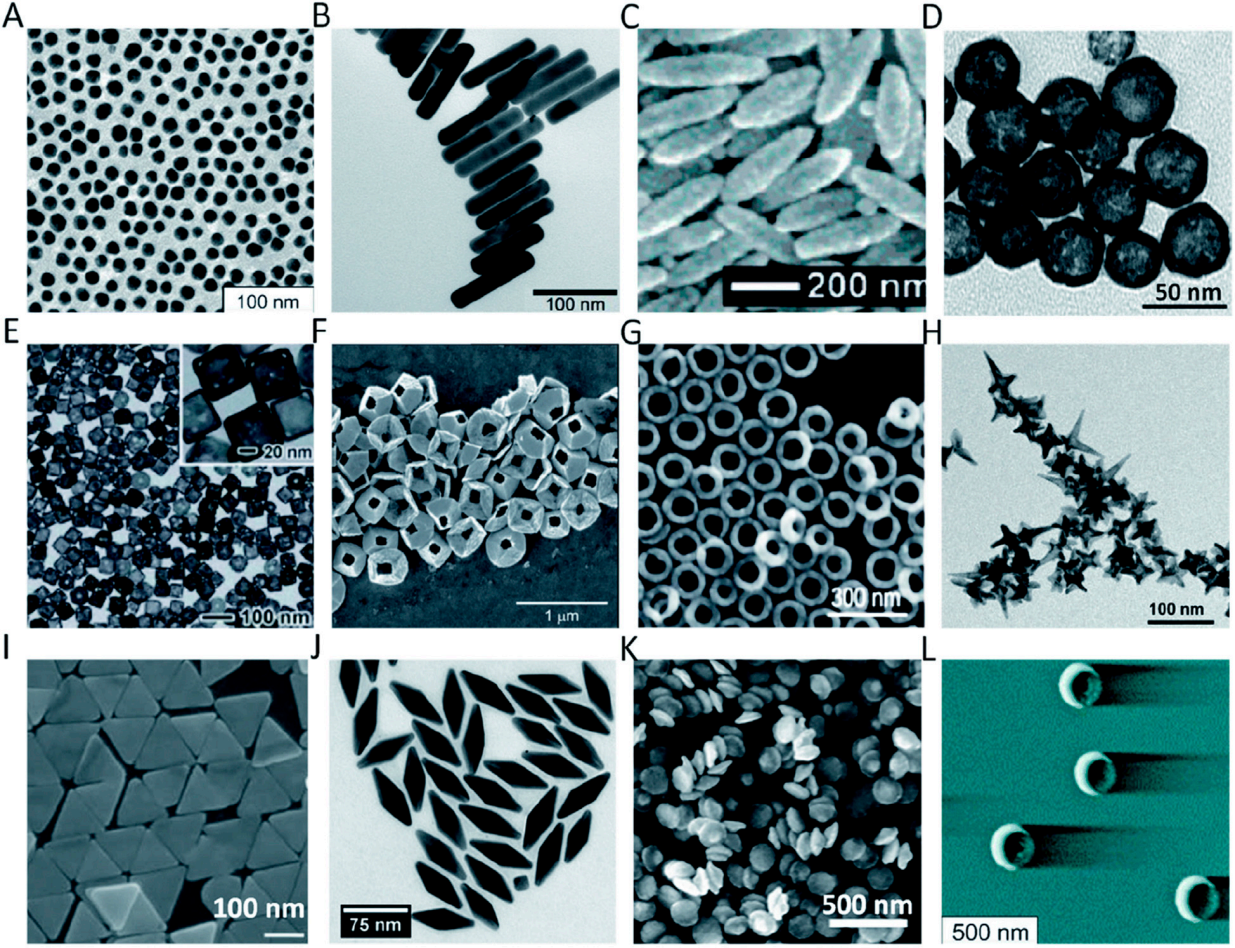
Different shapes and sizes of Au nanoparticles in bioimaging: **(A)** AuNS; **(B)** AuNR; **(C)** Au nanorice; **(D)** AuNSh; **(E)** AuNC; **(F)** tipless AuNPy; **(G)** AuNRg; **(H)** AuNSt; **(I)** AuNPr; **(J)** AuNBP; **(K)** AuND; and **(L)** AuNCr. Reproduction with the permission from reference (Si et al., 2021). Copyright ^©^ 2021 The Author(s).

Metal nanoparticles, particularly gold and silver nanoparticles, have attracted significant attention for biomedical imaging (Yoon et al., 2015; Dąbrowska-Bouta et al., 2018). Gold nanoparticles, due to their unique optical and electronic properties, are widely used in bioimaging (Si et al., 2021) and techniques like enhanced light scattering (Figure 1). These nanoparticles significantly improve imaging contrast and resolution through surface plasmon resonance (Kim et al., 2019). Their size and shape can be precisely controlled during synthesis to fine-tune their optical properties (Piella et al., 2016). For example, gold nanorods, because of their anisotropic shape, exhibit distinct longitudinal and transverse surface plasmon resonance peaks, enabling signal enhancement across different wavelengths (Zheng et al., 2021; Huang et al., 2009). Furthermore, the gold nanoparticle surface can be chemically modified with various functional groups for targeted delivery and specific molecular recognition.

Silver nanoparticles, known for their antibacterial properties and excellent optical characteristics (Fahmy et al., 2019; Singh et al., 2015; Balachandran et al., 2013), also hold great promise for biomedical imaging. Like gold nanoparticles, their size and shape can be precisely controlled during synthesis to adjust their optical properties (Shenashen et al., 2014; Pryshchepa et al., 2020; Fahmy et al., 2019). Silver nanoparticles significantly enhance sensitivity and resolution through surface-enhanced Raman scattering (SERS) (Seney et al., 2009; Zhang et al., 2011). SERS not only improves resolution but also provides detailed molecular information, potentially enabling earlier diagnosis of vascular diseases (Joseph et al., 2018).

### 2.2 Magnetic nanoparticles

**FIGURE 2:**
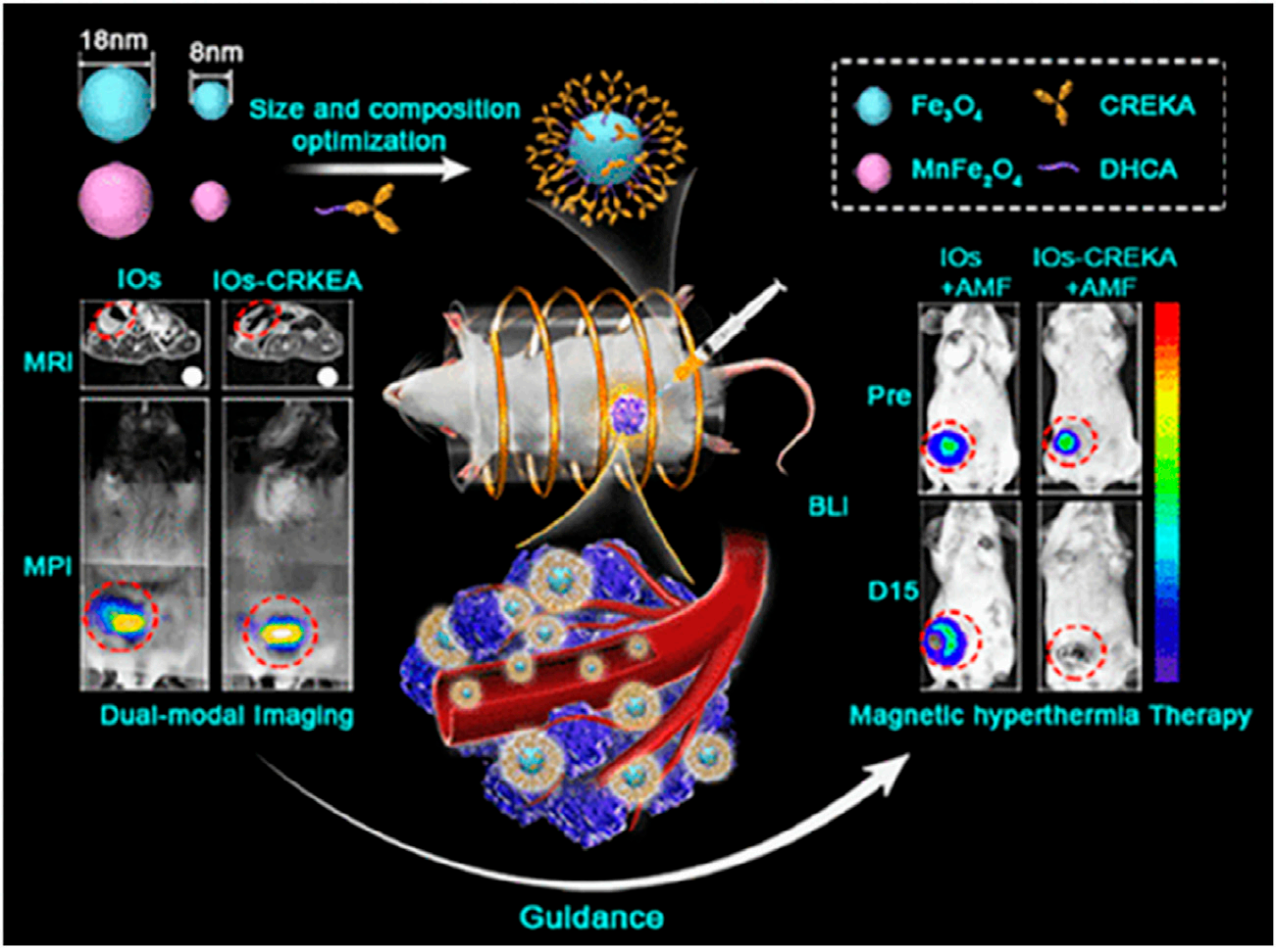
CREKA Modified Iron Oxide (IO) NPs for Precision Cancer Imaging and magnetic hyperthermia therapy. Reproduction with the permission from reference (Du et al., 2019). Copyright ^©^ 2019 American Chemical Society.

Magnetic nanoparticles are widely used in MRI (Du et al., 2019; Yang et al., 2010). These nanoparticles significantly enhance MRI imaging contrast and spatial resolution due to their strong magnetic properties (Figure 2). The core of magnetic nanoparticles is typically composed of magnetic materials, while the outer layer is coated with polymers or other materials to improve their biocompatibility and stability. In addition to enhancing MRI contrast, magnetic nanoparticles can be combined with other imaging techniques to develop multimodal imaging technologies. Such multimodal imaging technologies not only improve imaging sensitivity and specificity but also enable a comprehensive diagnosis of lesions through complementary imaging modes. Moreover, magnetic nanoparticles exhibit excellent magnetic thermal effects, generating local high temperatures under an external alternating magnetic field, thereby being used for hyperthermia of lesions (Prieto and Linares, 2018; Skandalakis et al., 2020). This multifunctional characteristic of magnetic nanoparticles shows great potential in cerebrovascular imaging and treatment.

### 2.3 Quantum dots

**FIGURE 3:**
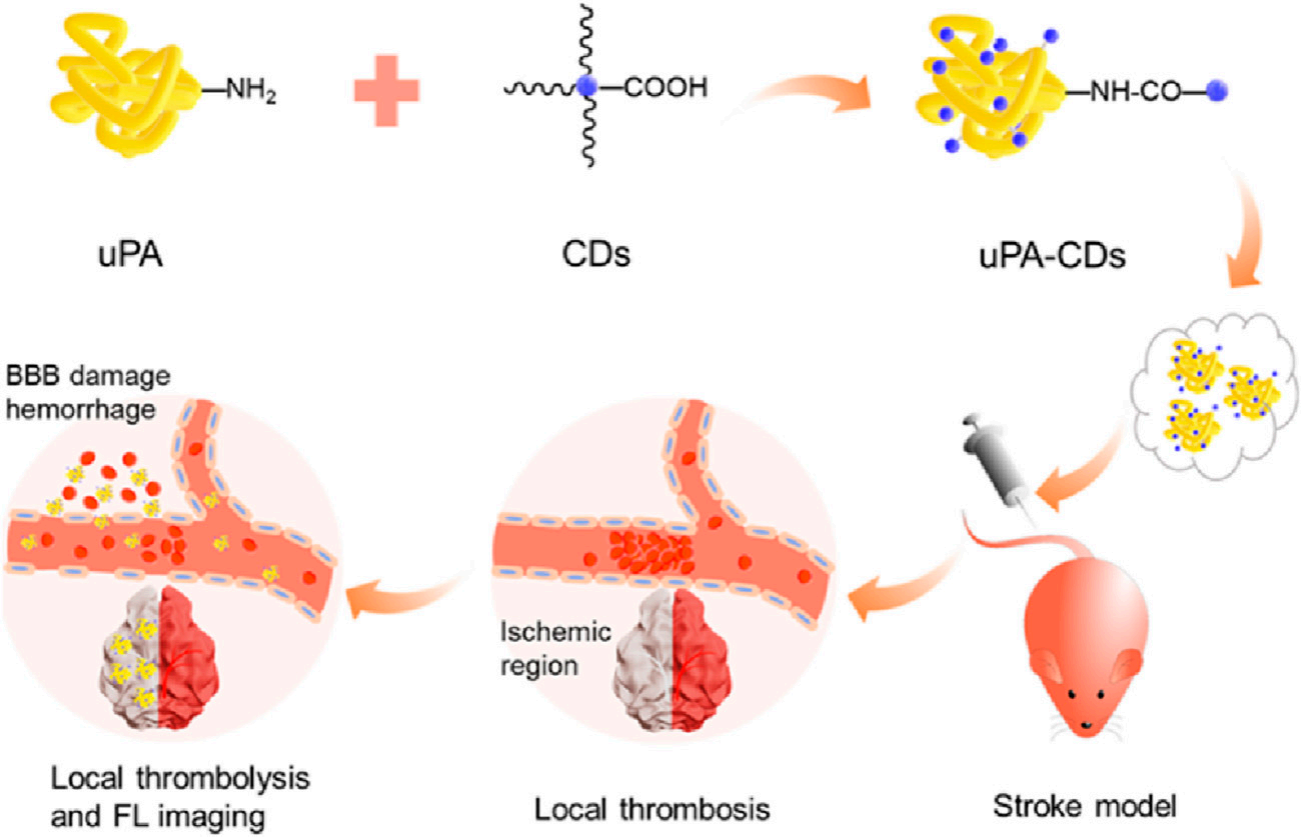
Urokinase-type plasminogen activator (uPA)-carbon dot (CD) Nanohybrids for fluorescence (FL) Imaging of Early blood−brain barrier (BBB) injury. Reproduction with the permission of reference (Niu et al., 2020). 2020. Copyright ^©^ 2020 American Chemical Society.

Quantum dots are nanocrystals made of semiconductor materials with unique optical and electronic properties, such as wide excitation spectra, narrow and symmetrical emission spectra, and high optical stability (Agarwal et al., 2023). These properties give quantum dots great potential in fluorescence imaging. Compared to traditional organic dyes, quantum dots offer higher brightness and longer fluorescence lifetime, enabling higher resolution and sensitivity imaging (Resch-Genger et al., 2008). Quantum dots can also be used for fluorescence imaging (Pandey and Bodas, 2020), where multiple quantum dots are simultaneously excited by different wavelengths of excitation light, displaying the distribution and dynamic changes of multiple biomolecules in the same image. This fluorescence imaging technology can provide rich spatial and temporal information at the molecular level, helping to reveal the complex biological mechanisms of cerebrovascular lesions (Niu et al., 2020) (Figure 3). Additionally, the spectral properties of quantum dots can be adjusted by changing their size and composition (Moreels et al., 2009), achieving fluorescence emission in different wavelength ranges. This flexibility broadens the application of quantum dots in cerebrovascular imaging (Ren et al., 2021), allowing for the design and optimization of imaging schemes based on specific needs.

### 2.4 Carbon-based nanomaterials

Carbon-based nanomaterials, such as graphene, carbon nanotubes, and fullerenes, show great potential in bioimaging due to their unique physical and chemical properties (Zhang et al., 2018). Graphene is a two-dimensional material composed of a single layer of carbon atoms with excellent electrical conductivity, mechanical strength, and thermal stability. In biomedical imaging, graphene and its derivatives (such as graphene oxide) can be used for multimodal imaging and photoacoustic imaging (Yang et al., 2012; Toumia et al., 2016) due to their unique optical and electrical properties. Photoacoustic imaging is a method that combines optical and ultrasonic technology, achieving high-resolution deep tissue imaging through the photoacoustic effect generated by laser excitation of graphene. The high thermal conductivity and absorption rate of graphene exhibit excellent signal enhancement effects in photoacoustic imaging, significantly improving the sensitivity and resolution of cerebrovascular imaging.

Carbon nanotubes are nanomaterials with a concentric cylindrical structure made of carbon atoms, exhibiting excellent mechanical properties and electrical conductivity (Manikandan et al., 2021). In cerebrovascular imaging, carbon nanotubes can be used as contrast agents or carriers to enhance imaging signals and target drug delivery (Loh et al., 2018). Additionally, the surface of carbon nanotubes can be functionalized with antibodies, peptides, or drug molecules to achieve targeted imaging and treatment of specific cerebrovascular regions or lesions. This multifunctional characteristic of carbon nanotubes provides a new approach for the diagnosis and treatment of cerebrovascular diseases.

Fullerenes are spherical nanomaterials composed of carbon atoms, possessing unique electronic and optical properties. In biomedical imaging, fullerenes can be used as photosensitizers or contrast agents to enhance imaging signals (Luo et al., 2021). Additionally, fullerenes exhibit excellent photodynamic effects, generating reactive oxygen species to induce cell apoptosis under light irradiation, thus being used for the cancer treatment (Li et al., 2024). This photodynamic therapy combined with imaging technology provides a new treatment approach for cerebrovascular diseases.

### 2.5 Polymer nanoparticles

Polymer nanoparticles are nanoparticles composed of biodegradable polymers, possessing good biocompatibility and flexibility. Polymer nanoparticles can be loaded with various imaging agents, such as fluorescent dyes, radioactive isotopes, or magnetic nanoparticles, to achieve multimodal imaging (Ong et al., 2021; Yu et al., 2022). Additionally, the surface of polymer nanoparticles can be functionalized with antibodies, peptides, or small molecule drugs to achieve targeted imaging and treatment of specific cerebrovascular regions or lesions (Gao et al., 2022). In addition to their application in imaging, polymer nanoparticles can also be used for drug delivery and gene therapy. For instance, polymer nanoparticles loaded with therapeutic drugs or gene vectors can achieve targeted delivery to cerebrovascular lesion sites (Luo et al., 2020; Zhang et al., 2022), improving treatment efficiency and reducing side effects. This multifunctional characteristic of polymer nanoparticles provides a new approach for the integrated diagnosis and treatment of cerebrovascular diseases.

## 3 Applications of nanomaterials in different imaging techniques

### 3.1 Magnetic resonance imaging (MRI)

**FIGURE 4:**
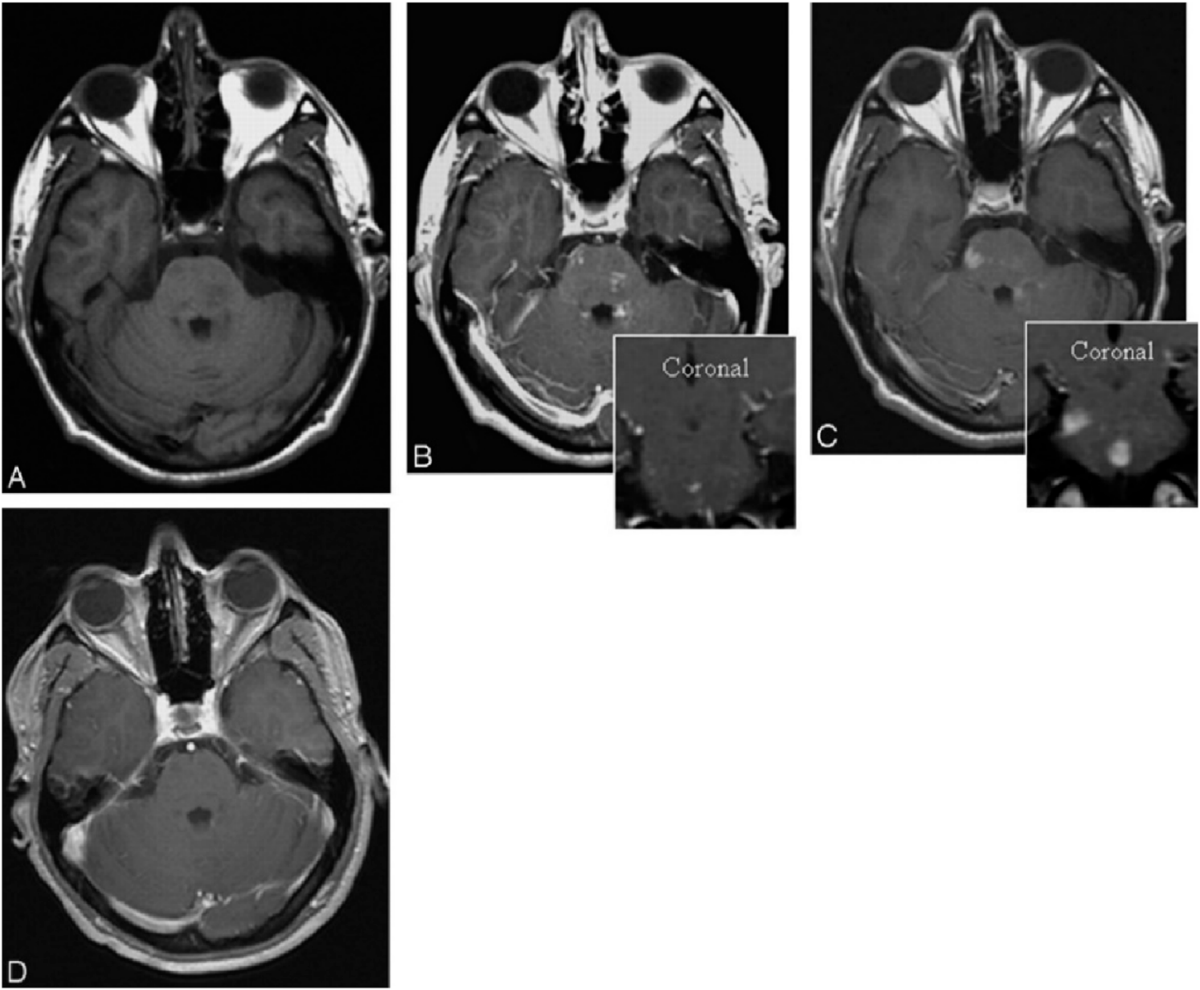
Patient with ADEM. Axial T1-weighted images without **(A)** and with **(B)** gadolinium (inset coronal) reveal faint and subtle enhancement in multiple brain stem lesions. Six days later **(C)**, the same site exhibits significant, more pronounced, and larger enhancement with ferumoxtran-10 (inset, coronal). Three months later **(D)**, the lesions no longer show enhancement on T1-weighted images with gadolinium.

**FIGURE 5:**
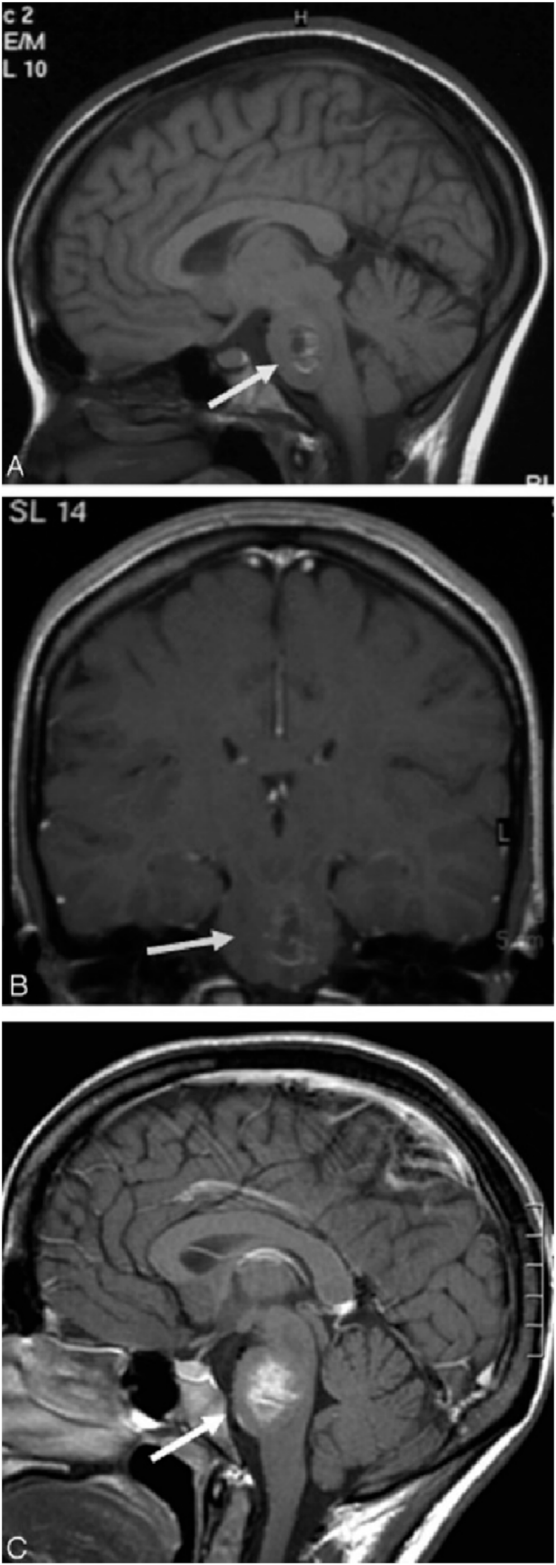
Patient with cavernous venous vascular malformation. Sagittal T1-weighted images without gadolinium **(A)** and coronal T1-weighted images with gadolinium **(B)** show no significant enhancement in the left pons lesion. However, 4 days later, T1 MR images **(C)** reveal prominent enhancement of the same lesion with ferumoxtran-10. Reproduction with the permission of reference (Manninger et al., 2005). Copyright ^©^2005 American Society of Neuroradiology.

**FIGURE 6:**
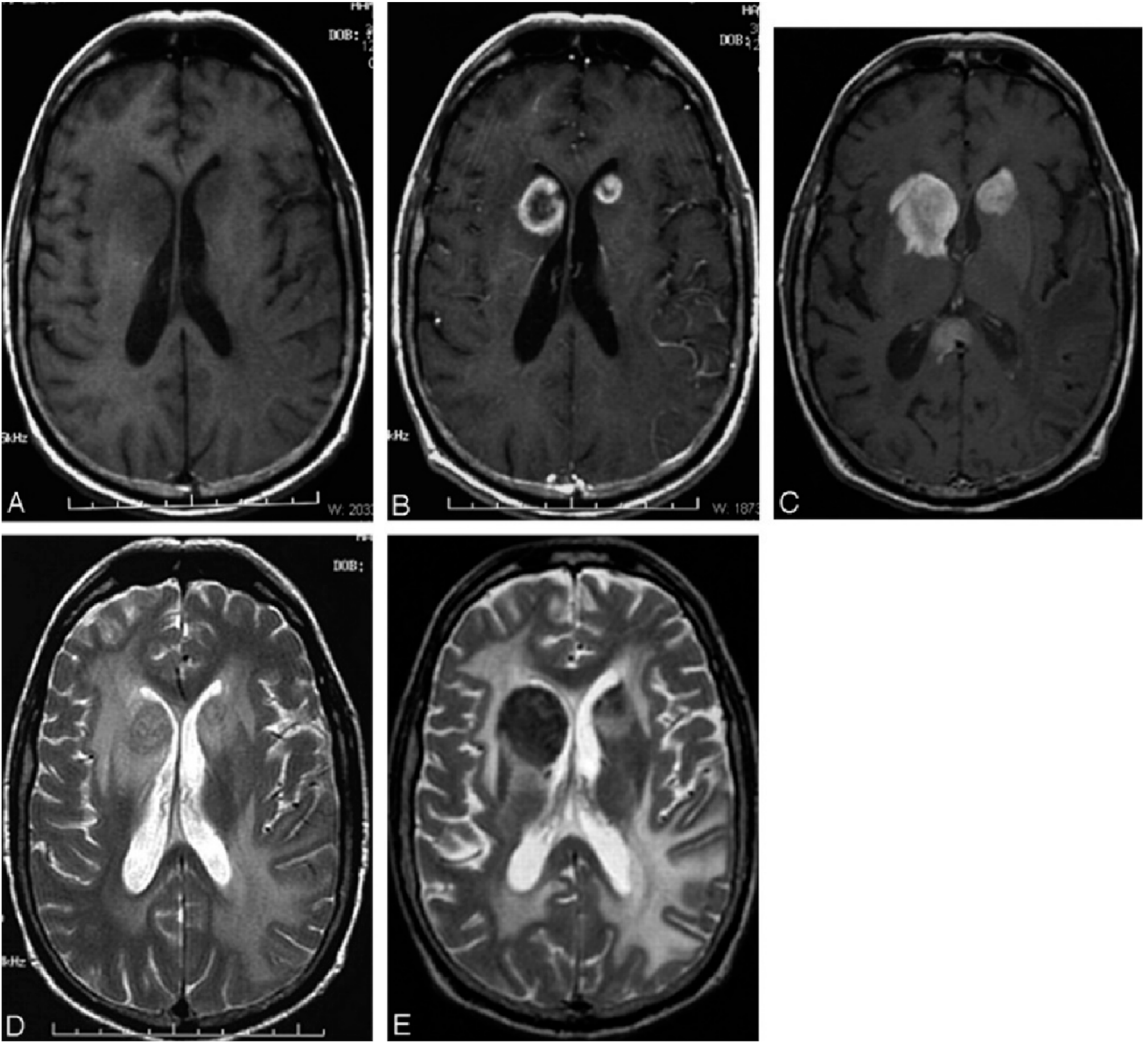
Patient with PCNSL. Axial T1-weighted images without gadolinium **(A)** and with gadolinium **(B)** reveal intense ring-enhancing lesions in the head of both caudate nuclei. Fifteen days later, T1-weighted images with ferumoxtran-10 **(C)** demonstrate that the lesions in the caudate head have enlarged and exhibit more intense enhancement. Additionally, another lesion in the splenium of the corpus callosum is visible, showing larger and more intense enhancement compared to the baseline gadolinium image (not shown). T2-weighted images following ferumoxtran-10 administration **(D, E)** display low-intensity changes in both lesions. Reproduction with the permission of reference (Manninger et al., 2005). Copyright ^©^2005 American Society of Neuroradiology Reproduction with the permission of reference (Manninger et al., 2005). Copyright ^©^ 2005 American Society of Neuroradiology.

Magnetic Resonance Imaging (MRI) holds a significant position in cerebrovascular imaging because it provides high-resolution soft tissue contrast without the use of ionizing radiation. Traditional MRI contrast agents are typically gadolinium-based compounds, but their potential nephrotoxicity limits their long-term and high-dose use. Iron oxide-based contrast agents have been investigated as more specific MR imaging agents for central nervous system (CNS) inflammation. Ferumoxtran-10 is a virus-sized nanoparticle, taken up by reactive cells, that allows visualization of the phagocytic components of CNS lesions. Manninger et al. compared Ferumoxtran-10 with standard gadolinium-enhanced MR images in an exploratory trial to assess its potential in the evaluation of CNS lesions with inflammatory aspects (Manninger et al., 2005). In this study, twenty-three patients with different types of intracranial “inflammatory” lesions underwent standard brain MR with and without gadolinium, followed an average of 10 days later by a ferumoxtran-10 scan. Patients were imaged 24 h after infusion of 2.6 mg/kg ferumoxtran-10. All MR images were evaluated subjectively by 4 investigators for a difference in enhancement patterns. The results showed that the ferumoxtran-10 scan showed higher signal intensity, larger area of enhancement, or new enhancing areas compared with gadolinium, indicating that Ferumoxtran-10 showed different enhancement patterns in a variety of CNS lesions with inflammatory components in comparison to gadolinium (Figures 4–6).

**FIGURE 7:**
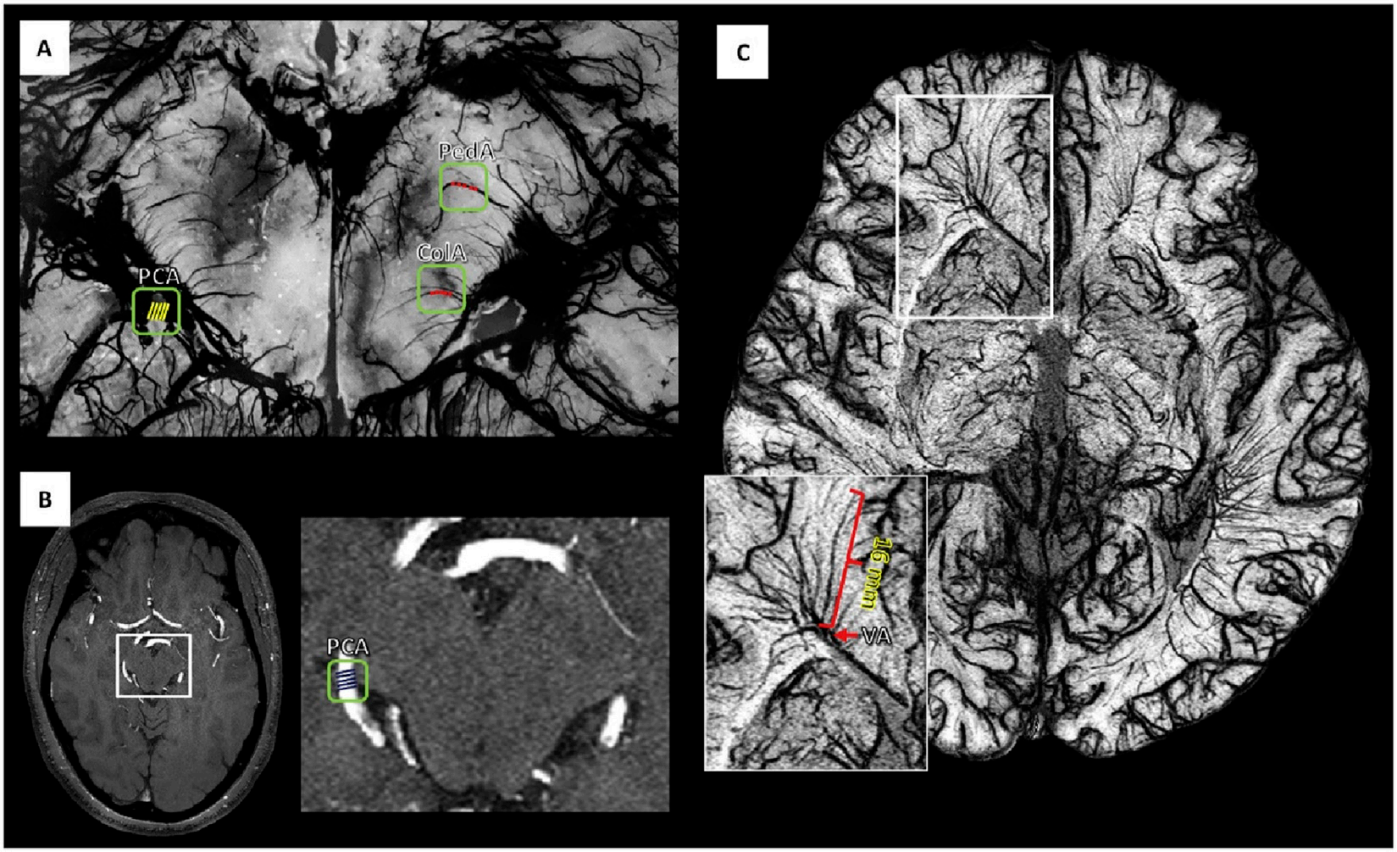
Validating the size of the small vessels that were visible after administration of USPIO agent Ferumoxytol **(A)** The ratio of posterior cerebral artery (PCA) to a peduncular artery (PedA) and collicular artery (ColA) was estimated using the histology work by obtaining the vessel diameters at five different locations. **(B)** The true diameter of PCA was obtained from Fe0 short TE magnitude data for all subjects. **(C)** SWIPGAC data (with a zoomed inset of the medullary veins as shown by the white box), focusing on the cerebral white matter vessels. VA = Ventricular angle. Reproduction with the permission of reference (Buch et al., 2020). Copyright © 2020 Elsevier Inc.

Advanced nanomaterials, especially magnetic nanoparticles like USPIO, offer an attractive alternative. USPIO exhibits excellent T2 relaxation effects, which can significantly enhance the contrast of T2-weighted MRI images (Figure 7). These nanoparticles enter the bloodstream through intravenous injection and can target and accumulate at cerebrovascular lesion sites, such as atherosclerotic plaques and tumor vasculature, thereby providing high-resolution cerebrovascular imaging (Buch et al., 2020).

The surface of magnetic nanoparticles can be functionalized to introduce various targeting molecules (such as antibodies, peptides, or small molecule drugs) to enhance their targeting specificity. For example, USPIO modified with anti-angiogenic factor antibodies can specifically bind to the neovasculature of brain tumors (Soliman et al., 2020; Abousalman‐Rezvani et al., 2024), enabling precise imaging of lesion sites. In addition to enhancing MRI contrast, magnetic nanoparticles can also be combined with other imaging techniques to develop multimodal imaging probes (Antonelli and Magnani, 2022). By conjugating USPIO with fluorescent dyes or radioactive isotopes, simultaneous imaging with MRI and fluorescence imaging or positron emission tomography (PET) can be achieved, thereby providing more comprehensive information on cerebrovascular lesions (Zhang et al., 2023; Yu et al., 2018).

### 3.2 Computed tomography (CT)

Computed Tomography (CT) is widely used in cerebrovascular imaging, especially in the diagnosis of acute stroke (Mangesius et al., 2021). However, traditional iodine-based contrast agents have limited effectiveness in CT imaging and pose potential nephrotoxicity risks (Zhang P. et al., 2021). Advanced nanomaterials, particularly gold nanoparticles, offer new possibilities for enhancing CT imaging. Gold nanoparticles have a high atomic number and density, which significantly increases X-ray absorption, thereby enhancing CT imaging contrast (Aslan et al., 2020; Asadinezhad et al., 2021). Compared to traditional contrast agents, gold nanoparticles have a longer circulation time in the bloodstream, providing more sustained imaging effects (Owens et al., 2023).

The size and shape of gold nanoparticles can be adjusted during the synthesis process to optimize their performance in CT imaging (Piella et al., 2016). For instance, spherical and rod-shaped gold nanoparticles have different optical and physical properties, making them suitable for various imaging applications (Alam et al., 2022; Bansal et al., 2020). Surface-functionalized gold nanoparticles can achieve targeted imaging of specific cerebrovascular lesion sites through targeting molecules (such as antibodies or peptides). For example, gold nanoparticles modified with anti-platelet-derived growth factor (PDGF) antibodies can target the vasculature of brain tumors, providing high-resolution CT images (Meola et al., 2018).

Besides enhancing CT imaging, gold nanoparticles can also be combined with other imaging modalities to develop multifunctional imaging probes. For example, conjugating gold nanoparticles with fluorescent dyes or magnetic nanoparticles allows for simultaneous CT and fluorescence imaging or MRI (Cheng et al., 2014). This multimodal imaging technology can provide more comprehensive information on lesions, aiding in the improved diagnosis and treatment of cerebrovascular diseases. For instance, the combination of gold nanoparticles and magnetic nanoparticles enables both CT and MRI imaging (Cheng et al., 2014; Sun et al., 2016), offering detailed images of cerebrovascular structures and functions.

Despite the promising prospects of gold nanoparticles in CT imaging, their biosafety and long-term *in vivo* metabolism require further research. Studies have shown that the surface modification of gold nanoparticles can significantly affect their distribution and metabolic pathways in the body. For example, polyethylene glycol (PEG) modification can increase the blood circulation time of gold nanoparticles and reduce their accumulation in the liver and spleen (Kozics et al., 2021), thereby enhancing biosafety. Future research should focus on optimizing the surface modification and functionalization of gold nanoparticles to improve their feasibility and safety in clinical applications.

### 3.3 Fluorescence imaging

**FIGURE 8:**
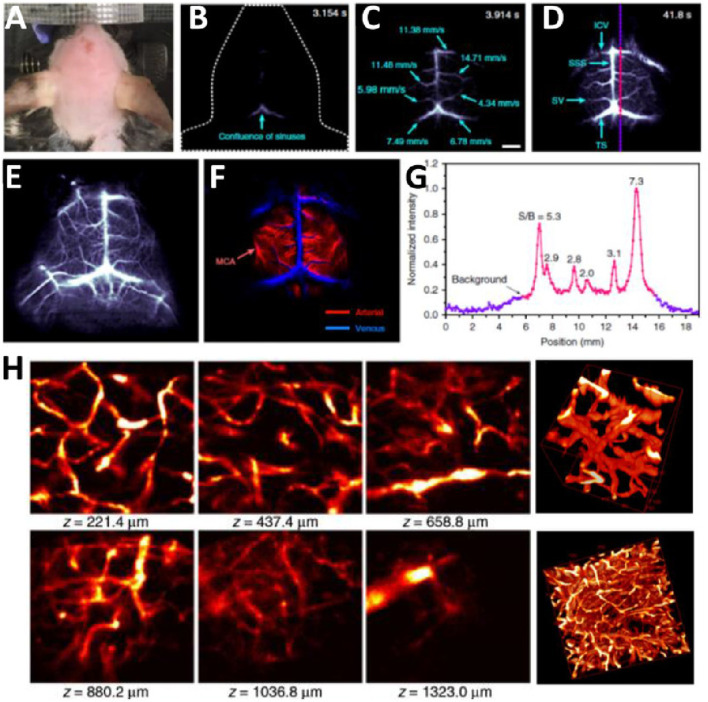
**(A)** Color photograph of a hair-shaved-off C57BL/6 mouse for NIR-IIb fluorescence imaging. **(B–D)** Time-course NIR-IIb brain fluorescence images present the perfusion of rare-earth NPs into various cerebral vessels. **(E, F)** Cerebral vascular image and the corresponding PCA overlaid image F showing arterial (red) and venous (blue) vessels. **(G)** SBR analysis of NIR-IIb cerebrovascular image. **(H)** Small area and 3D reconstruction of vascular confocal images of a mouse brain. Reproduction with the permission of reference (Ren et al., 2021). Copyright © 2021 The Authors.

Fluorescence imaging technology has significant applications in biomedical research and clinical diagnostics, particularly in cerebrovascular imaging (Ren et al., 2021), where it provides high sensitivity and high-resolution real-time imaging (Figure 8). Quantum dots, as an advanced fluorescent probe material, have become a research hotspot in fluorescence imaging due to their unique optical properties and high brightness. Quantum dots have broad excitation and narrow emission spectra, enabling the simultaneous excitation of multiple colors for multicolor imaging (Wang et al., 2020). In cerebrovascular imaging, quantum dots can achieve highly specific imaging of cerebrovascular lesion sites through surface modification with specific targeting molecules. For example, quantum dots conjugated with vascular endothelial growth factor (VEGF) antibodies can target and image areas of neovascularization in the brain (Li et al., 2015), providing strong support for the early diagnosis of brain tumors and strokes. The high optical stability of quantum dots allows them to maintain brightness without fading during prolonged imaging, providing continuous, clear images (Montalti et al., 2015). This is important for dynamically monitoring the progression of cerebrovascular lesions and the effectiveness of treatments. Additionally, the emission spectra of quantum dots can be tuned by adjusting their size and composition (Hu et al., 2015), allowing fluorescence emission over different wavelength ranges and enabling multicolor imaging and multiparameter analysis.

The application of quantum dots in cerebrovascular imaging also includes multimodal imaging (Jing et al., 2014; Swierczewska et al., 2011). By combining quantum dots with magnetic nanoparticles or metal nanoparticles, it is possible to achieve multimodal imaging with fluorescence imaging, magnetic resonance imaging (MRI), and computed tomography (CT). This multimodal imaging technology can provide comprehensive information about lesions, including structural, functional, and molecular-level data, thereby improving diagnostic accuracy and treatment effectiveness. For example, composite probes combining quantum dots and magnetic nanoparticles can switch between fluorescence imaging and MRI (Koole et al., 2009), achieving high-sensitivity, multidimensional imaging of cerebrovascular lesions.

Despite the significant advantages of quantum dots in fluorescence imaging, their biosafety remains a concern. Quantum dots often contain heavy metal elements, such as cadmium and lead, which can cause toxic effects when accumulated in the body. Therefore, developing low-toxicity or non-toxic quantum dot materials, such as carbon quantum dots or silicon quantum dots, is a current research focus. By optimizing the surface modification and functionalization of quantum dots, their biocompatibility and *in vivo* stability can be further enhanced, promoting their development in clinical applications. For instance, coating quantum dots with biocompatible polymer materials can significantly reduce their toxicity and improve their stability and distribution specificity *in vivo*.

### 3.4 Multimodal imaging

Multimodal imaging technology holds significant importance in cerebrovascular imaging as it combines the advantages of various imaging modalities, providing more comprehensive and detailed information about lesions. Advanced nanomaterials open up new possibilities for the development of multimodal imaging probes. For instance, combining metal nanoparticles with fluorescent dyes, magnetic nanoparticles, or radioactive isotopes can enable the simultaneous application of multiple imaging modes, such as fluorescence imaging, magnetic resonance imaging (MRI), computed tomography (CT), and positron emission tomography (PET) (Huang and Davis, 2011; Burke et al., 2017).

Metal nanoparticles, such as gold and silver nanoparticles, are widely used in multimodal imaging. Gold nanoparticles, with their unique surface plasmon resonance (SPR) properties, can significantly enhance optical imaging contrast. Additionally, their high atomic number makes them excellent at X-ray absorption, thus providing high-contrast CT images. Combining gold nanoparticles with fluorescent dyes allows for simultaneous fluorescence and CT imaging. For example, gold nanoparticles modified with vascular endothelial growth factor (VEGF) antibodies can be used for both fluorescence and CT imaging, providing detailed information about neovascularization in brain tumors (Hariharan et al., 2022).

Magnetic nanoparticles, such as USPIO, also have significant advantages in multimodal imaging. USPIO exhibits excellent T2 relaxation effects, significantly enhancing the contrast of T2-weighted MRI images. By combining USPIO with fluorescent dyes or radioactive isotopes, it is possible to achieve simultaneous MRI and fluorescence imaging or PET imaging (Chen et al., 2016). For instance, composite probes combining USPIO and fluorescent dyes can switch between MRI and fluorescence imaging, providing high-sensitivity, multidimensional imaging of cerebrovascular lesions (Deddens et al., 2012). Moreover, the magnetic hyperthermia effect of USPIO can be used for brain tumor hyperthermia treatment (Israel et al., 2020), offering a non-invasive localized therapy option in addition to imaging.

**FIGURE 9:**
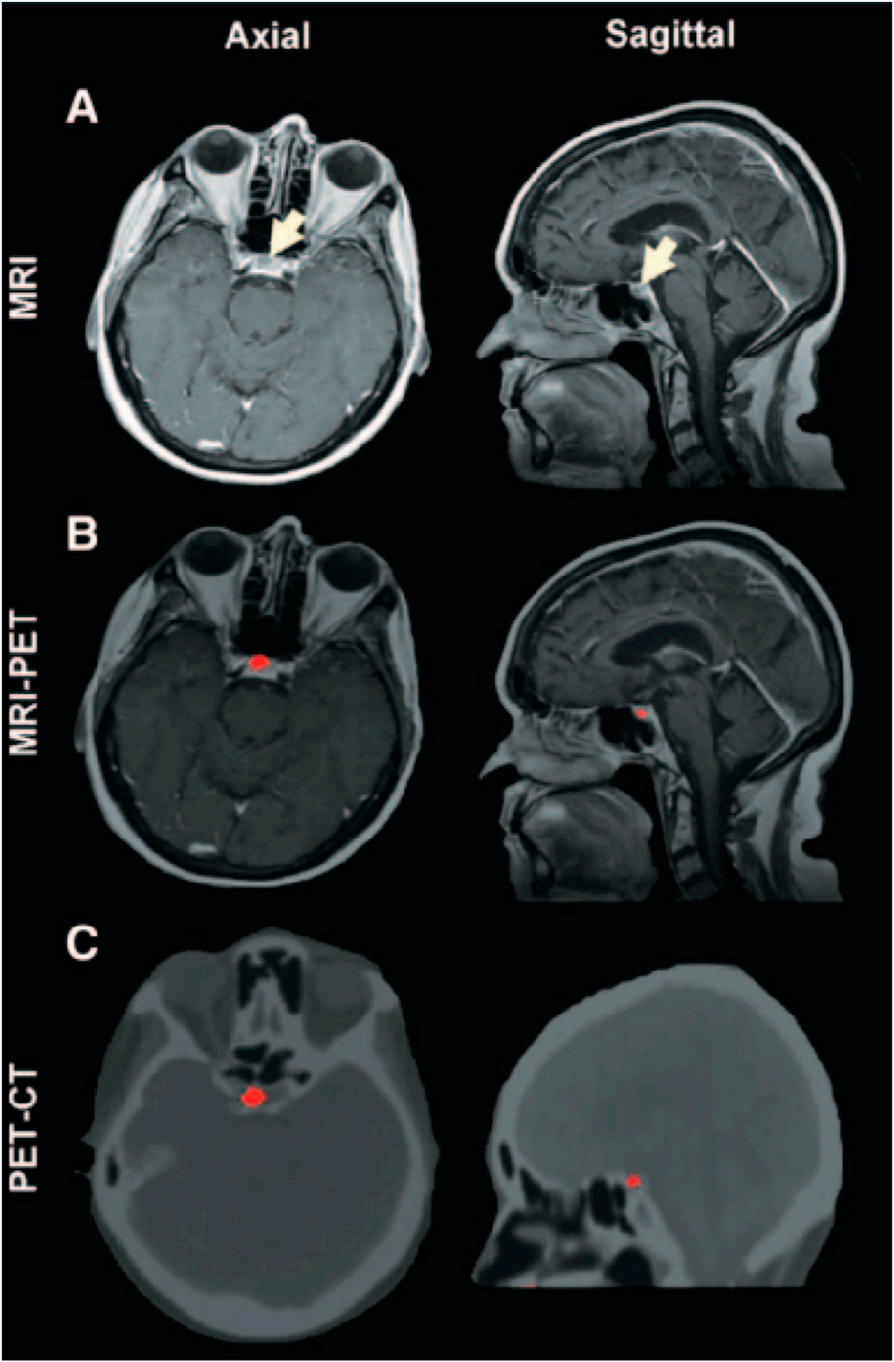
Multimodal Imaging of Particle Uptake in a Pituitary Lesion. **(A)** Multiplanar contrast-enhanced MR axial and sagittal images of patient, taken 72 h post-injection, demonstrate a subcentimeter cystic focus (arrows) within the right aspect of the anterior pituitary gland. **(B)** Co-registered axial and sagittal MRI-PET images show increased focal activity (red, 124I-cRGDY–PEG–C dots) localized to the lesion site. **(C)** Axial and sagittal PET-CT images further localize this activity to the right aspect of the sella. Reproduction with the permission of reference (Phillips et al., 2014). Copyright ^©^2014 American Association for the Advancement of Science.

Carbon-based nanomaterials, such as graphene and carbon nanotubes, also show great potential in multimodal imaging (Yang et al., 2012). Graphene, with its excellent optical, electrical, and mechanical properties, is particularly suitable for photoacoustic imaging (Toumia et al., 2016). Combining graphene with fluorescent dyes or magnetic nanoparticles can achieve multimodal imaging with photoacoustic imaging, fluorescence imaging, and MRI. For example, composite probes combining graphene and magnetic nanoparticles can switch between photoacoustic imaging and MRI, providing high-resolution structural and functional images of cerebrovascular structures (Zhao et al., 2021). Carbon nanotubes, with their unique structure and optical properties, can be utilized to develop multifunctional imaging probes capable of simultaneous fluorescence, photoacoustic, and CT imaging (Thangam et al., 2021). Phillips et al. conducted a human clinical trial involving ultrasmall inorganic hybrid nanoparticles, specifically ^124^I-cRGDY–PEG–C dot particles, in patients (Phillips et al., 2014). In patient who received an intravenous injection of ^124^I-cRGDY–PEG–C dots, a well-defined cystic lesion approximately 5 mm in size was observed in the right anterior lobe of the pituitary gland, an area lacking a blood-brain barrier, on axial and sagittal magnetic resonance (MR) images (Figure 9A). Precise co-registration of this tracer-avid focus with multiplanar MRI (Figure 9B) and CT (Figure 9C) images confirmed its location within the interior of the pituitary gland. PET imaging revealed a progressive net accumulation of ^124^I-cRGDY–PEG–C dot activity at the lesion site (Figure 9D).

Polymer nanoparticles also deserve attention in multimodal imaging applications (Joshi and Joshi, 2022). Polymer nanoparticles have good biocompatibility and degradability, allowing them to carry various imaging probes or drugs for efficient targeted delivery and imaging. For example, polylactic-co-glycolic acid (PLGA) nanoparticles can carry fluorescent dyes, magnetic nanoparticles, or drug molecules for multimodal imaging and targeted therapy (Hashemi et al., 2020; Zhang et al., 2020). By combining PLGA nanoparticles with metal or magnetic nanoparticles, it is possible to develop multimodal imaging probes with fluorescence, MRI, and photoacoustic imaging capabilities (Joshi and Joshi, 2022). This multimodal imaging technology can provide more comprehensive and detailed information about cerebrovascular lesions, thus improving diagnostic accuracy and treatment effectiveness.

The main advantage of multimodal imaging lies in its ability to integrate the benefits of different imaging modalities, providing comprehensive information at structural, functional, and molecular levels. For example, MRI can offer high-resolution soft tissue contrast, CT can provide high-contrast images of bones and blood vessels, fluorescence imaging can deliver highly sensitive molecular information, and PET can offer metabolic and functional data. By combining these imaging modalities, it is possible to obtain more comprehensive information about lesions, thereby improving diagnostic accuracy and treatment outcomes. In brain tumor diagnosis, for instance, multimodal imaging can provide multidimensional information about tumor anatomy, angiogenesis, molecular marker expression, and metabolic activity, offering more comprehensive diagnostic support for clinicians (Overcast et al., 2021).

In summary, the application of advanced nanomaterials in cerebrovascular imaging has broad prospects. By continuously optimizing and improving the synthesis processes and surface modification techniques of nanomaterials, their imaging performance and biosafety can be further enhanced, promoting their translation and application in clinical settings. The use of multimodal imaging technology, particularly in combination with different types of nanomaterials, offers new approaches and methods for the diagnosis and treatment of cerebrovascular diseases. In the future, as nanomaterials and imaging technologies continue to advance, multimodal imaging will play an increasingly important role in cerebrovascular imaging, providing strong support for the early diagnosis and personalized treatment of cerebrovascular diseases.

## 4 Challenges and future prospects

Despite the significant advantages of nanomaterials in cerebrovascular imaging, there are still many challenges and issues in practical applications. For example, the biosafety, long-term stability, targeting, specificity, large-scale preparation, and clinical translation of nanomaterials require further research and solutions. Therefore, future research should focus on the following aspects.

### 4.1 Biosafety and long-term stability

A primary challenge for using advanced nanomaterials in cerebrovascular imaging is ensuring their safety. This involves both biocompatibility and minimizing potential toxicity. Nanomaterials have unique properties ideal for imaging, but these same properties can raise safety concerns. Some nanomaterials, like quantum dots and certain metallic nanoparticles, contain potentially toxic elements (e.g., cadmium, lead) (Sengul and Asmatulu, 2020; Sobhanan et al., 2023). These elements can accumulate in the body, harming tissues and organs. Therefore, biocompatibility is crucial for clinical translation. Nanomaterial toxicity is linked to several factors: size, shape, surface chemistry, and functional modifications (Sukhanova et al., 2018; Albanese et al., 2012; Kim et al., 2013). Smaller nanoparticles can more easily cross biological barriers, potentially increasing their biological impact and toxicity (Pandey and Prajapati, 2018; Sani et al., 2021). Functional modifications, such as coating with polyethylene glycol (PEG), can improve biocompatibility. PEGylation can extend blood circulation time and reduce accumulation in the liver and spleen, minimizing toxic reactions (Fan et al., 2020). Metabolism and excretion pathways are also critical for biosafety. Ideally, nanomaterials should be biodegradable and quickly cleared from the body to prevent long-term accumulation and toxicity. Some polymer-based nanoparticles (e.g., PLGA) show promise in this regard, breaking down into harmless molecules. However, further research is needed to optimize their degradation and metabolic profiles. Researchers are developing strategies to enhance biocompatibility. Surface coating with biocompatible materials (e.g., liposomes, proteins, polysaccharides) can improve stability and reduce toxicity. Liposome-coated gold nanoparticles, for example, show improved blood stability and reduced accumulation in organs (Liang et al., 2017).

In conclusion, while biocompatibility and toxicity are key challenges, ongoing research into surface modifications and other strategies offers promising solutions. Rigorous testing and clinical trials are essential to validate the safety and efficacy of these nanomaterials before widespread use in cerebrovascular imaging.

### 4.2 Targeting and specificity

Targeting and specificity are crucial for effective cerebrovascular imaging using nanomaterials. These materials must accurately reach and accumulate at the lesion site to generate clear imaging signals. However, the blood-brain barrier (BBB) hinders most nanomaterials from entering brain tissue, limiting their application. Therefore, developing nanomaterials capable of crossing the BBB and targeting cerebrovascular lesions is a key research area (Furtado et al., 2018; Tan et al., 2023; Zhang W. et al., 2021; Gao et al., 2022; Wu et al., 2023). Several strategies enhance targeting. Surface functionalization with ligands (e.g., antibodies, peptides, small molecules) allows nanoparticles to bind specifically to target sites, such as the neovasculature of brain tumors (Winer et al., 2011). Multifunctional molecules can simultaneously provide targeting and imaging capabilities, improving specificity and sensitivity (Rizvi et al., 2021). Leveraging the physicochemical properties of nanomaterials is another approach. Magnetic nanoparticles (e.g., USPIO) can be guided to specific locations using an external magnetic field, improving targeting efficiency and reducing non-specific distribution in healthy tissues, which also lowers potential toxicity (Liu et al., 2021). Nanomaterial size and shape also influence targeting. Non-spherical shapes (e.g., rods, sheets) interact differently with blood vessels compared to spheres, potentially improving accumulation at lesion sites. Precise control over size and shape can optimize *in vivo* distribution and targeting (Annu et al., 2022; Luo et al., 2020).

Despite these advances, challenges remain. The BBB’s complexity and individual variability hinder penetration and targeting. The body’s complex metabolism and clearance mechanisms can also reduce effectiveness. Future research should explore new targeting strategies and optimize existing technologies to enhance nanomaterial performance in cerebrovascular imaging. This includes further investigation into overcoming the BBB and improving the precision of targeting to specific lesion types within the cerebrovascular system.

### 4.3 Large-scale preparation and clinical translation

Using nanomaterials for cerebrovascular imaging depends on high-quality materials and scalable production. Optimized preparation processes are essential for consistent material performance. Traditional methods (e.g., chemical precipitation, sol-gel, hydrothermal synthesis, sputtering) (Rafique et al., 2020) can produce high-quality nanomaterials at small scales, but scaling up often presents challenges like low yield, high cost, and complex processes. Newer techniques aim to improve production efficiency and quality. Microfluidic technology (Zhao et al., 2020; Illath et al., 2022), using microscale reactors, allows for precise control over reaction conditions. This leads to greater material uniformity and stability, shorter preparation times, and reduced costs. It also facilitates continuous synthesis of multi-component nanomaterials, enabling the development of multifunctional imaging probes. Self-assembly is another promising technique (Li et al., 2022). By controlling intermolecular forces (e.g., electrostatic, hydrogen bonds, van der Waals), nanomaterials can spontaneously assemble into complex structures. This method is efficient, environmentally friendly, and potentially scalable. It allows for the creation of multilayer nanomaterials capable of carrying multiple imaging probes or drugs for multimodal imaging and targeted therapy (Li et al., 2021).

Despite these advances, large-scale production still faces challenges. Maintaining consistent product quality and reproducibility requires stable process parameters and stringent quality control. Surface modification and functionalization also add complexity. Addressing these challenges requires optimizing process parameters, developing automated equipment, and implementing robust quality control systems. Researchers are exploring new processes and equipment to improve large-scale production. High-pressure homogenizers and ultrasonic dispersers enhance homogenization and dispersion, preventing agglomeration and ensuring product stability. Continuous flow reactors and automated control systems can further increase efficiency and production capacity. Cost-effectiveness and environmental impact are also important considerations. Traditional methods often use large amounts of solvents and energy, creating waste. Developing green, sustainable processes, such as aqueous phase synthesis (using water as a solvent), is crucial for reducing costs and environmental impact.

In summary, efficient preparation and scalable production are critical for translating nanomaterials to clinical cerebrovascular imaging. Optimizing existing methods, developing new technologies and equipment, and prioritizing green chemistry are key areas for future research. These efforts will improve the availability and cost-effectiveness of nanomaterials for clinical use.

### 4.4 Regulatory and ethical issues

Nanomaterials in cerebrovascular imaging face not only scientific and technical hurdles but also regulatory and ethical challenges. As nanotechnology rapidly advances, governments and international organizations are developing regulations and guidelines to ensure safety, efficacy, and responsible use. Nanomaterials must meet stringent requirements for clinical trials and commercialization. Regulatory processes for nanomaterials are similar to those for traditional drugs and medical devices, but with added complexities. The unique properties of nanomaterials may require new evaluation standards and approval procedures. Regulatory agencies typically require comprehensive safety and toxicity data to assess potential human health and environmental impacts. Clear standards and guidelines are also needed for material preparation, quality control, and clinical use.

Ethical considerations are equally important. While nanomaterials offer great potential for improved diagnostics and treatment, their long-term effects remain uncertain. Potential risks, such as organ damage or immune responses from long-term accumulation, require careful monitoring and evaluation. Balancing scientific progress with individual rights is also crucial. This includes ensuring informed consent for patients in clinical trials, protecting patient rights, and promoting equitable access to nanomaterial-based technologies. Open discussions among scientists, medical institutions, governments, and the public are essential for developing appropriate guidelines and policies.

In conclusion, addressing regulatory and ethical issues is essential for the successful translation of nanomaterials to cerebrovascular imaging. Establishing robust regulatory frameworks, unified safety standards, and clear ethical guidelines will ensure responsible development and application of these promising technologies. Continued dialogue and collaboration are crucial for maximizing the benefits of nanomaterials while minimizing potential risks.

## 5 Conclusion

The rapid development of nanotechnology has brought new opportunities to cerebrovascular imaging. Advanced nanomaterials, such as metal nanoparticles, magnetic nanoparticles, quantum dots, carbon-based nanomaterials, and polymer nanoparticles, have shown great potential in improving imaging resolution, contrast, and specificity. These nanomaterials improve imaging sensitivity and specificity through various physical and chemical mechanisms, providing new approaches for the diagnosis and treatment of cerebrovascular diseases. Despite the significant advantages of nanomaterials, there are still many challenges and issues in practical applications, such as biosafety, long-term stability, targeting, specificity, large-scale preparation, and clinical translation. Therefore, future research should focus on addressing these issues to promote the further development and application of advanced nanomaterials in cerebrovascular imaging. Through in-depth discussion of these issues, this review hopes to provide valuable references for researchers in related fields and promote the further development and application of advanced nanomaterials in cerebrovascular imaging.

## References

[B1] Abbas S. G. Kumar S. (2018). Importance of computed tomography scan in patients with cerebrovascular accidents. J. Adv. Med. Dent. Sci. Res. 6 (11), 48.

[B2] Abousalman‐Rezvani Z. Refaat A. Dehghankelishadi P. Roghani‐Mamaqani H. Esser L. Voelcker N. H. (2024). Insights into targeted and stimulus‐responsive nanocarriers for brain cancer treatment. Adv. Healthc. Mater. 13 (12), 2302902. 10.1002/adhm.202302902 38199238

[B3] Agarwal K. Rai H. Mondal S. (2023). Quantum dots: an overview of synthesis, properties, and applications. Mater. Res. Express 10 (6), 062001. 10.1088/2053-1591/acda17

[B4] Alam Md. S. Farhad S. F. U. Tanvir N. I. Bitu Md. N. A. Mohammad M. Mahmuda H. (2022) “Spherical and rod-shaped gold nanoparticles for surface enhanced Raman spectroscopy,” in 2022 4th international conference on sustainable technologies for industry 4.0 (STI). IEEE, 1–4.

[B5] Albanese A. Tang P. S. Chan W. C. W. (2012). The effect of nanoparticle size, shape, and surface chemistry on biological systems. Annu. Rev. Biomed. Eng. 14, 1–16. 10.1146/annurev-bioeng-071811-150124 22524388

[B6] Altammar K. A. (2023). A review on nanoparticles: characteristics, synthesis, applications, and challenges. Front. Microbiol. 14, 1155622. 10.3389/fmicb.2023.1155622 37180257 PMC10168541

[B7] Anderson D. Anderson T. Fahmi F. (2019). Advances in applications of metal oxide nanomaterials as imaging contrast agents. Phys. status solidi (a) 216 (16), 1801008. 10.1002/pssa.201801008

[B8] Annu S. A. Qamar Z. Md S. Alhakamy N. A. Baboota S. Ali J. (2022). An insight to brain targeting utilizing polymeric nanoparticles: effective treatment modalities for neurological disorders and brain tumor. Front. Bioeng. Biotechnol. 10, 788128. 10.3389/fbioe.2022.788128 35186901 PMC8851324

[B9] Antonelli A. Magnani M. (2022). SPIO nanoparticles and magnetic erythrocytes as contrast agents for biomedical and diagnostic applications. J. magnetism magnetic Mater. 541, 168520. 10.1016/j.jmmm.2021.168520

[B10] Asadinezhad M. Azimian H. Ghadiri H. Khademi S. (2021). Gold nanoparticle parameters play an essential role as CT imaging contrast agents. J. Nanostructures 11 (4), 668–677. 10.22052/JNS.2021.04.005

[B11] Aslan N. Ceylan B. Koç M. M. Findik F. (2020). Metallic nanoparticles as X-Ray computed tomography (CT) contrast agents: a review. J. Mol. Struct. 1219, 128599. 10.1016/j.molstruc.2020.128599

[B12] Balachandran Y. L. Girija S. Selvakumar R. Tongpim S. Gutleb A. C. Suriyanarayanan S. (2013). Differently environment stable bio-silver nanoparticles: study on their optical enhancing and antibacterial properties. PLoS One 8 (10), e77043. 10.1371/journal.pone.0077043 24130832 PMC3793943

[B13] Bansal S. A. Kumar V. Karimi J. Singh A. P. (2020). Role of gold nanoparticles in advanced biomedical applications. Nanoscale Adv. 2 (9), 3764–3787. 10.1039/d0na00472c 36132791 PMC9419294

[B14] Buch S. Wang Y. Park M. G. Jella P. K. Hu J. Chen Y. (2020). Subvoxel vascular imaging of the midbrain using USPIO-Enhanced MRI. Neuroimage 220, 117106. 10.1016/j.neuroimage.2020.117106 32615253 PMC9401191

[B15] Burke B. P. Cawthorne C. Archibald S. J. (2017). Multimodal nanoparticle imaging agents: design and applications. Philosophical Trans. R. Soc. A Math. Phys. Eng. Sci. 375 (2107), 20170261. 10.1098/rsta.2017.0261 29038384

[B16] Campos E. V. R. Pereira A. E. S. De Oliveira J. L. Carvalho L. B. Guilger-Casagrande M. de Lima R. (2020). How can nanotechnology help to combat COVID-19? Opportunities and urgent need. J. Nanobiotechnology 18, 125. 10.1186/s12951-020-00685-4 32891146 PMC7474329

[B17] Cardoso V. F. Francesko A. Ribeiro C. Bañobre‐López M. Martins P. Lanceros‐Mendez S. (2018). Advances in magnetic nanoparticles for biomedical applications. Adv. Healthc. Mater. 7 (5), 1700845. 10.1002/adhm.201700845 29280314

[B18] Chen D. Dougherty C. A. Yang D. Wu H. Hong H. (2016). Radioactive nanomaterials for multimodality imaging. Tomography 2 (1), 3–16. 10.18383/j.tom.2016.00121 27227167 PMC4876975

[B19] Cheng Y. Morshed R. A. Auffinger B. Tobias A. L. Lesniak M. S. (2014). Multifunctional nanoparticles for brain tumor imaging and therapy. Adv. drug Deliv. Rev. 66, 42–57. 10.1016/j.addr.2013.09.006 24060923 PMC3948347

[B20] Chung S. Revia R. A. Zhang M. (2021). Graphene quantum dots and their applications in bioimaging, biosensing, and therapy. Adv. Mater. 33 (22), 1904362. 10.1002/adma.201904362 PMC7289657 31833101

[B21] Dąbrowska-Bouta B. Sulkowski G. Frontczak-Baniewicz M. Skalska J. Sałek M. Orzelska-Górka J. (2018). Ultrastructural and biochemical features of cerebral microvessels of adult rat subjected to a low dose of silver nanoparticles. Toxicology 408, 31–38. 10.1016/j.tox.2018.06.009 29935189

[B22] Deddens L. H. Van Tilborg G. A. F. Mulder W. J. M. De Vries H. E. Dijkhuizen R. M. (2012). Imaging neuroinflammation after stroke: current status of cellular and molecular MRI strategies. Cerebrovasc. Dis. 33 (4), 392–402. 10.1159/000336116 22456323

[B23] Du Y. Liu X. Liang Q. Tian J. (2019). Optimization and design of magnetic ferrite nanoparticles with uniform tumor distribution for highly sensitive MRI/MPI performance and improved magnetic hyperthermia therapy. Nano Lett. 19 (6), 3618–3626. 10.1021/acs.nanolett.9b00630 31074627

[B24] Elsabahy M. Wooley K. L. (2012). Design of polymeric nanoparticles for biomedical delivery applications. Chem. Soc. Rev. 41 (7), 2545–2561. 10.1039/c2cs15327k 22334259 PMC3299918

[B25] Fahmy H. M. Mosleh A. M. Abd Elghany A. Shams-Eldin E. Abu Serea E. S. Ali S. A. (2019). Coated silver nanoparticles: synthesis, cytotoxicity, and optical properties. RSC Adv. 9 (35), 20118–20136. 10.1039/c9ra02907a 35514687 PMC9065456

[B26] Fakruddin M. Hossain Z. Afroz H. (2012). Prospects and applications of nanobiotechnology: a medical perspective. J. nanobiotechnology 10, 31–38. 10.1186/1477-3155-10-31 22817658 PMC3422163

[B27] Fan Z. Zhu P. Zhu Y. Wu K. Li C. Y. Cheng H. (2020). Engineering long-circulating nanomaterial delivery systems. Curr. Opin. Biotechnol. 66, 131–139. 10.1016/j.copbio.2020.07.006 32795661

[B28] Furtado D. Björnmalm M. Ayton S. Bush A. I. Kempe K. Caruso F. (2018). Overcoming the blood–brain barrier: the role of nanomaterials in treating neurological diseases. Adv. Mater. 30 (46), 1801362. 10.1002/adma.201801362 30066406

[B29] Gao X. Xu J. Yao T. Liu X. Zhang H. Zhan C. (2022). Peptide-decorated nanocarriers penetrating the blood-brain barrier for imaging and therapy of brain diseases. Adv. drug Deliv. Rev. 187, 114362. 10.1016/j.addr.2022.114362 35654215

[B30] Ghekiere O. Salgado R. Buls N. Leiner T. Mancini I. Vanhoenacker P. (2017). Image quality in coronary CT angiography: challenges and technical solutions. Br. J. radiology 90 (1072), 20160567. 10.1259/bjr.20160567 PMC5605061 28055253

[B31] Gollavelli G. Ghule A. V. Ling Y. C. (2022). Multimodal imaging and phototherapy of cancer and bacterial infection by graphene and related nanocomposites. Molecules 27 (17), 5588. 10.3390/molecules27175588 36080351 PMC9457605

[B32] Granqvist C. G. Buhrman R. A. (1976). Ultrafine metal particles. J. Appl. Phys. 47 (5), 2200–2219. 10.1063/1.322870

[B33] Hage Z. A. Alaraj A. Arnone G. D. Charbel F. T. (2016). Novel imaging approaches to cerebrovascular disease. Transl. Res. 175, 54–75. 10.1016/j.trsl.2016.03.018 27094991

[B34] Haller S. Vernooij M. W. Kuijer J. P. A. Larsson E. M. Jäger H. R. Barkhof F. (2018). Cerebral microbleeds: imaging and clinical significance. Radiology 287 (1), 11–28. 10.1148/radiol.2018170803 29558307

[B35] Hariharan K. Parekh K. Rani M. Mehta T. (2022) “Development and applications of gold nanoparticles for targeting brain tumors,” in Nanocarriers for drug-targeting brain tumors. Elsevier, 485–512.

[B36] Hashemi M. Shamshiri A. Saeedi M. Tayebi L. Yazdian-Robati R. (2020). Aptamer-conjugated PLGA nanoparticles for delivery and imaging of cancer therapeutic drugs. Archives Biochem. biophysics 691, 108485. 10.1016/j.abb.2020.108485 32712288

[B37] Haupt K. Medina Rangel P. X. Bui B. T. S. (2020). Molecularly imprinted polymers: antibody mimics for bioimaging and therapy. Chem. Rev. 120 (17), 9554–9582. 10.1021/acs.chemrev.0c00428 32786424

[B38] Hu S. Trinchi A. Atkin P. Cole I. (2015). Tunable photoluminescence across the entire visible spectrum from carbon dots excited by white light. Angew. Chem. Int. Ed. 54 (10), 2970–2974. 10.1002/anie.201411004 25589468

[B39] Huang W. Y. Davis J. J. (2011). Multimodality and nanoparticles in medical imaging. Dalton Trans. 40 (23), 6087–6103. 10.1039/c0dt01656j 21409202 PMC4498419

[B40] Huang X. Neretina S. El‐Sayed M. A. (2009). Gold nanorods: from synthesis and properties to biological and biomedical applications. Adv. Mater. 21 (48), 4880–4910. 10.1002/adma.200802789 25378252

[B41] Illath K. Kar S. Gupta P. Shinde A. Wankhar S. Tseng F. G. (2022). Microfluidic nanomaterials: from synthesis to biomedical applications. Biomaterials 280, 121247. 10.1016/j.biomaterials.2021.121247 34801251

[B42] Israel L. Galstyan A. Holler E. Ljubimova J. Y. (2020). Magnetic iron oxide nanoparticles for imaging, targeting and treatment of primary and metastatic tumors of the brain. J. Control. Release 320, 45–62. 10.1016/j.jconrel.2020.01.009 31923537 PMC7641100

[B43] Jeon M. Halbert M. V. Stephen Z. R. Zhang M. (2021). Iron oxide nanoparticles as T1 contrast agents for magnetic resonance imaging: fundamentals, challenges, applications, and prospectives. Adv. Mater. 33 (23), 1906539. 10.1002/adma.201906539 PMC8022883 32495404

[B44] Jing L. Ding K. Kershaw S. V. Kempson I. M. Rogach A. L. Gao M. (2014). Magnetically engineered semiconductor quantum dots as multimodal imaging probes. Adv. Mater. 26 (37), 6367–6386. 10.1002/adma.201402296 25178258

[B45] Joseph M. M. Narayanan N. Nair J. B. Karunakaran V. Ramya A. N. Sujai P. T. (2018). Exploring the margins of SERS in practical domain: an emerging diagnostic modality for modern biomedical applications. Biomaterials 181, 140–181. 10.1016/j.biomaterials.2018.07.045 30081304

[B46] Joshi B. Joshi A. (2022). Polymeric magnetic nanoparticles: a multitargeting approach for brain tumour therapy and imaging. Drug Deliv. Transl. Res. 12, 1588–1604. 10.1007/s13346-021-01063-9 34537930

[B47] Kim J. Lee S.-K. Schellingerhout D. Nahrendorf M. Kim K. Kim J. (2019). Spectroscopic assessment of gold nanoparticle biodistribution using surface plasmon resonance phenomena. ACS Biomaterials Sci. and Eng. 5 (12), 6389–6394. 10.1021/acsbiomaterials.9b01079 33417791

[B48] Kim S. T. Saha K. Kim C. Rotello V. M. (2013). The role of surface functionality in determining nanoparticle cytotoxicity. Accounts Chem. Res. 46 (3), 681–691. 10.1021/ar3000647 PMC3640732 23294365

[B49] Koole R. Mulder W. J. M. Van Schooneveld M. M. Strijkers G. J. Meijerink A. Nicolay K. (2009). Magnetic quantum dots for multimodal imaging. Wiley Interdiscip. Rev. Nanomedicine Nanobiotechnology 1 (5), 475–491. 10.1002/wnan.14 20049812

[B50] Kozics K. Sramkova M. Kopecka K. Begerova P. Manova A. Krivosikova Z. (2021). Pharmacokinetics, biodistribution, and biosafety of PEGylated gold nanoparticles *in vivo* . Nanomaterials 11 (7), 1702. 10.3390/nano11071702 34203551 PMC8305691

[B51] Kumar H. Venkatesh N. Bhowmik H. Kuila A. (2018). Metallic nanoparticle: a review. Biomed. J. Sci. Tech. Res. 4 (2), 3765–3775. 10.26717/bjstr.2018.04.0001011

[B52] Li C. Cao L. Zhang Y. Yi P. Wang M. Tan B. (2015). Preoperative detection and intraoperative visualization of brain tumors for more precise surgery: a new dual‐modality MRI and NIR nanoprobe. small 11 (35), 4517–4525. 10.1002/smll.201500997 26058947

[B53] Li C. Shuford K. L. Park Q. H. Cai W. Li Y. Lee E. (2007). High-yield synthesis of single-crystalline gold nano-octahedra. Angew. Chemie-International Ed. 46 (18), 3264–3268. 10.1002/anie.200604167 17387671

[B54] Li L. Fu J. Ye J. Liu L. Sun Z. Wang H. (2024). Developing hypoxia‐sensitive system via designing tumor‐targeted fullerene‐based photosensitizer for multimodal therapy of deep tumor. Adv. Mater. 36 (23), 2310875. 10.1002/adma.202310875 38450765

[B55] Li S. Wei J. Yao Q. Song X. Xie J. Yang H. (2023). Emerging ultrasmall luminescent nanoprobes for *in vivo* bioimaging. Chem. Soc. Rev. 52 (5), 1672–1696. 10.1039/d2cs00497f 36779305

[B56] Li T. Lu X. M. Zhang M. R. Hu K. Li Z. (2022). Peptide-based nanomaterials: self-assembly, properties and applications. Bioact. Mater. 11, 268–282. 10.1016/j.bioactmat.2021.09.029 34977431 PMC8668426

[B57] Li Z. Fan Q. Yin Y. (2021). Colloidal self-assembly approaches to smart nanostructured materials. Chem. Rev. 122 (5), 4976–5067. 10.1021/acs.chemrev.1c00482 34747588

[B58] Liang G. Wang H. Shi H. Wang H. Zhu M. Jing A. (2020). Recent progress in the development of upconversion nanomaterials in bioimaging and disease treatment. J. Nanobiotechnology 18, 154. 10.1186/s12951-020-00713-3 33121496 PMC7596946

[B59] Liang R. Xie J. Li J. Wang K. Liu L. Gao Y. (2017). Liposomes-coated gold nanocages with antigens and adjuvants targeted delivery to dendritic cells for enhancing antitumor immune response. Biomaterials 149, 41–50. 10.1016/j.biomaterials.2017.09.029 28992509

[B60] Lin Y. Zhang K. Zhang R. She Z. Tan R. Fan Y. (2020). Magnetic nanoparticles applied in targeted therapy and magnetic resonance imaging: crucial preparation parameters, indispensable pre-treatments, updated research advancements and future perspectives. J. Mater. Chem. B 8 (28), 5973–5991. 10.1039/d0tb00552e 32597454

[B61] Liu X. Zhang H. Zhang T. Wang Y. Jiao W. Lu X. (2021). Magnetic nanomaterials-mediated cancer diagnosis and therapy. Prog. Biomed. Eng. 4 (1), 012005. 10.1088/2516-1091/ac3111 41287228

[B62] Loh K. P. Ho D. Chiu G. N. C. Leong D. T. Pastorin G. Chow E. K. (2018). Clinical applications of carbon nanomaterials in diagnostics and therapy. Adv. Mater. 30 (47), 1802368. 10.1002/adma.201802368 30133035

[B63] Lu X. Y. Wu D. C. Li Z. J. Chen G. Q. (2011). Polymer nanoparticles. Prog. Mol. Biol. Transl. Sci. 104, 299–323. 10.1016/b978-0-12-416020-0.00007-3 22093222

[B64] Luo Y. Wang Y. Fu J. (2021). Nanomaterials in cerebrovascular disease diagnose and treatment. Part. and Part. Syst. Charact. 38 (5), 2000311. 10.1002/ppsc.202000311

[B65] Luo Y. Yang H. Zhou Y. F. Hu B. (2020). Dual and multi-targeted nanoparticles for site-specific brain drug delivery. J. Control. release 317, 195–215. 10.1016/j.jconrel.2019.11.037 31794799

[B66] Mangesius S. Janjic T. Steiger R. Haider L. Rehwald R. Knoflach M. (2021). Dual-energy computed tomography in acute ischemic stroke: state-of-the-art. Eur. Radiol. 31, 4138–4147. 10.1007/s00330-020-07543-9 33319330 PMC8128835

[B67] Manikandan N. Vp S. K. Rathis G. G R. T.k S. (2021). Carbon nanotubes and their properties-The review. Mater. today Proc. 47, 4682–4685. 10.1016/j.matpr.2021.05.543

[B68] Manninger S. P. Muldoon L. L. Nesbit G. Murillo T. Jacobs P. M. Neuwelt E. A. (2005). An exploratory study of ferumoxtran-10 nanoparticles as a blood-brain barrier imaging agent targeting phagocytic cells in CNS inflammatory lesions. Am. J. Neuroradiol. 26 (9), 2290–2300. 16219835 PMC7976152

[B69] Meola A. Rao J. Chaudhary N. Sharma M. Chang S. D. (2018). Gold nanoparticles for brain tumor imaging: a systematic review. Front. neurology 9, 328. 10.3389/fneur.2018.00328 PMC5960696 29867737

[B70] Montalti M. Cantelli A. Battistelli G. (2015). Nanodiamonds and silicon quantum dots: ultrastable and biocompatible luminescent nanoprobes for long-term bioimaging. Chem. Soc. Rev. 44 (14), 4853–4921. 10.1039/c4cs00486h 26051500

[B71] Moreels I. Lambert K. Smeets D. De Muynck D. Nollet T. Martins J. C. (2009). Size-dependent optical properties of colloidal PbS quantum dots. ACS nano 3 (10), 3023–3030. 10.1021/nn900863a 19780530

[B72] Hosseini S. M. Mohammadnejad J. Najafi-Taher R. Beiram Zadeh Z. Tanhaei M. Ramakrishna S. (2024). Multifunctional carbon-based nanoparticles. Theranostic applications in cancer therapy and diagnosis 10.1021/acsabm.2c01000 36921253

[B73] Niu Y. Tan H. Li X. Zhao L. Xie Z. Zhang Y. (2020). Protein–carbon dot nanohybrid-based early blood–brain barrier damage theranostics. ACS Appl. Mater. and interfaces 12 (3), 3445–3452. 10.1021/acsami.9b19378 31922399

[B74] Ong S. Y. Zhang C. Dong X. Yao S. Q. (2021). Recent advances in polymeric nanoparticles for enhanced fluorescence and photoacoustic imaging. Angew. Chem. Int. Ed. 60 (33), 17797–17809. 10.1002/anie.202101964 33709554

[B75] Orru’ E. Chung C. Y. Hui F. K. (2020). Cerebral Angiography. In: Neurointensive Care Unit. Current Clinical Neurology. Editor Nelson S. Nyquist P. (Cham: Humana).

[B76] Overcast W. B. Davis K. M. Ho C. Y. Hutchins G. D. Green M. A. Graner B. D. (2021). Advanced imaging techniques for neuro-oncologic tumor diagnosis, with an emphasis on PET-MRI imaging of malignant brain tumors. Curr. Oncol. Rep. 23, 34–15. 10.1007/s11912-021-01020-2 33599882 PMC7892735

[B77] Owens T. C. Anton N. Attia M. F. (2023). CT and X-ray contrast agents: current clinical challenges and the future of contrast. Acta Biomater. 171, 19–36. 10.1016/j.actbio.2023.09.027 37739244

[B78] Padmanabhan P. Kumar A. Kumar S. Chaudhary R. K. Gulyás B. (2016). Nanoparticles in practice for molecular-imaging applications: an overview. Acta biomater. 41, 1–16. 10.1016/j.actbio.2016.06.003 27265153

[B79] Pandey R. K. Prajapati V. K. (2018). Molecular and immunological toxic effects of nanoparticles. Int. J. Biol. Macromol. 107, 1278–1293. 10.1016/j.ijbiomac.2017.09.110 29017884

[B80] Pandey S. Bodas D. (2020). High-quality quantum dots for multiplexed bioimaging: a critical review. Adv. Colloid Interface Sci. 278, 102137. 10.1016/j.cis.2020.102137 32171116

[B81] Patel K. D. Singh R. K. Kim H. W. (2019). Carbon-based nanomaterials as an emerging platform for theranostics. Mater. Horizons 6 (3), 434–469. 10.1039/c8mh00966j

[B82] Phillips E. Penate-Medina O. Zanzonico P. B. Carvajal R. D. Mohan P. Ye Y. (2014). Clinical translation of an ultrasmall inorganic optical-PET imaging nanoparticle probe. Sci. Transl. Med. 6 (260), 260ra149. 10.1126/scitranslmed.3009524 PMC4426391 25355699

[B83] Piella J. Bastus N. G. Puntes V. (2016). Size-controlled synthesis of sub-10-nanometer citrate-stabilized gold nanoparticles and related optical properties. Chem. Mater. 28 (4), 1066–1075. 10.1021/acs.chemmater.5b04406

[B84] Prieto C. Linares I. (2018). Nanoparticles and nanothermia for malignant brain tumors, a suggestion of treatment for further investigations. Rep. Pract. Oncol. Radiotherapy 23 (5), 474–480. 10.1016/j.rpor.2018.08.001 PMC6158037 30263017

[B85] Pryshchepa O. Pomastowski P. Buszewski B. (2020). Silver nanoparticles: synthesis, investigation techniques, and properties. Adv. Colloid Interface Sci. 284, 102246. 10.1016/j.cis.2020.102246 32977142

[B86] Rafique M. S. Rafique M. Tahir M. B. Hajra S. Nawaz T. Shafiq F. (2020). Synthesis methods of nanostructures nanotechnology and photocatalysis for environmental applications. Elsevier, 45–56.

[B87] Ren F. Jiang Z. Han M. Zhang H. Yun B. Zhu H. (2021). NIR‐II Fluorescence imaging for cerebrovascular diseases. View 2 (6), 20200128. 10.1002/viw.20200128

[B88] Resch-Genger U. Grabolle M. Cavaliere-Jaricot S. Nitschke R. Nann T. (2008). Quantum dots versus organic dyes as fluorescent labels. Nat. methods 5 (9), 763–775. 10.1038/nmeth.1248 18756197

[B89] Reshma V. G. Mohanan P. V. (2019). Quantum dots: applications and safety consequences. J. Luminescence 205, 287–298. 10.1016/j.jlumin.2018.09.015

[B90] Rizvi S. F. A. Ali A. Ahmad M. Mu S. Zhang H. (2021). Multifunctional self-assembled peptide nanoparticles for multimodal imaging-guided enhanced theranostic applications against glioblastoma multiforme. Nanoscale Adv. 3 (20), 5959–5967. 10.1039/d1na00597a 36132681 PMC9419261

[B91] Sammet S. (2016). Magnetic resonance safety. Abdom. Radiol. 41, 444–451. 10.1007/s00261-016-0680-4 PMC4848040 26940331

[B92] Sani A. Cao C. Cui D. (2021). Toxicity of gold nanoparticles (AuNPs): a review. Biochem. biophysics Rep. 26, 100991. 10.1016/j.bbrep.2021.100991 PMC8063742 33912692

[B93] Seney C. S. Gutzman B. M. Goddard R. H. (2009). Correlation of size and surface-enhanced Raman scattering activity of optical and spectroscopic properties for silver nanoparticles. J. Phys. Chem. C 113 (1), 74–80. 10.1021/jp805698e

[B94] Sengul A. B. Asmatulu E. (2020). Toxicity of metal and metal oxide nanoparticles: a review. Environ. Chem. Lett. 18 (5), 1659–1683. 10.1007/s10311-020-01033-6

[B95] Shaban S. Huasen B. Haridas A. Killingsworth M. Worthington J. Jabbour P. (2022). Digital subtraction angiography in cerebrovascular disease: current practice and perspectives on diagnosis, acute treatment and prognosis. Acta Neurol. Belg. 122, 763–780. 10.1007/s13760-021-01805-z 34553337

[B96] Shen C. L. Liu H. R. Lou Q. Wang F. Liu K. K. Dong L. (2022). Recent progress of carbon dots in targeted bioimaging and cancer therapy. Theranostics 12 (6), 2860–2893. 10.7150/thno.70721 35401835 PMC8965501

[B97] Shenashen M. A. El‐Safty S. A. Elshehy E. A. (2014). Synthesis, morphological control, and properties of silver nanoparticles in potential applications. Part. and Part. Syst. Charact. 31 (3), 293–316. 10.1002/ppsc.201300181

[B98] Si P. Razmi N. Nur O. Solanki S. Pandey C. M. Gupta R. K. (2021). Gold nanomaterials for optical biosensing and bioimaging. Nanoscale Adv. 3 (10), 2679–2698. 10.1039/d0na00961j 36134176 PMC9418567

[B99] Sindhwani S. Chan W. C. W. (2021). Nanotechnology for modern medicine: next step towards clinical translation. J. Intern. Med. 290 (3), 486–498. 10.1111/joim.13254 33480120

[B100] Singh S. Bharti A. Meena V. K. (2015). Green synthesis of multi-shaped silver nanoparticles: optical, morphological and antibacterial properties. J. Mater. Sci. Mater. Electron. 26 (6), 3638–3648. 10.1007/s10854-015-2881-y

[B101] Skandalakis G. P. Rivera D. R. Rizea C. D. Bouras A. Jesu Raj J. G. Bozec D. (2020). Hyperthermia treatment advances for brain tumors. Int. J. Hyperth. 37 (2), 3–19. 10.1080/02656736.2020.1772512 PMC7756245 32672123

[B102] Sleight E. Stringer M. S. Marshall I. Wardlaw J. M. Thrippleton M. J. (2021). Cerebrovascular reactivity measurement using magnetic resonance imaging: a systematic review. Front. Physiology 12, 643468. 10.3389/fphys.2021.643468 PMC7947694 33716793

[B103] Sobhanan J. Rival J. V. Anas A. Sidharth Shibu E. Takano Y. Biju V. (2023). Luminescent quantum dots: synthesis, optical properties, bioimaging and toxicity. Adv. Drug Deliv. Rev. 197, 114830. 10.1016/j.addr.2023.114830 37086917

[B104] Soliman M. A. Guccione J. Reiter A. M. Moawad A. W. Etchison A. Kamel S. (2020). Current concepts in multi-modality imaging of solid tumor angiogenesis. Cancers 12 (11), 3239. 10.3390/cancers12113239 33153067 PMC7692820

[B105] Srinoi P. Chen Y. T. Vittur V. Marquez M. D. Lee T. R. (2018). Bimetallic nanoparticles: enhanced magnetic and optical properties for emerging biological applications. Appl. Sci. 8 (7), 1106. 10.3390/app8071106

[B106] Srivastava R. Thakur M. Kumawat M. K. Bahadur R. (2021). Graphene nanomaterials for multi-modal bioimaging and diagnosis of cancer. In: Next Generation Graphene Nanomaterials for Cancer Theranostic Applications. Singapore: Springer.

[B107] Sukhanova A. Bozrova S. Sokolov P. Berestovoy M. Karaulov A. Nabiev I. (2018). Dependence of nanoparticle toxicity on their physical and chemical properties. Nanoscale Res. Lett. 13, 44–21. 10.1186/s11671-018-2457-x 29417375 PMC5803171

[B108] Sun L. Joh D. Y. Al-Zaki A. Stangl M. Murty S. Davis J. J. (2016). Theranostic application of mixed gold and superparamagnetic iron oxide nanoparticle micelles in glioblastoma multiforme. J. Biomed. Nanotechnol. 12 (2), 347–356. 10.1166/jbn.2016.2173 27305768 PMC4942305

[B109] Swierczewska M. Lee S. Chen X. (2011). Inorganic nanoparticles for multimodal molecular imaging. Mol. Imaging 10 (1), 3–16. 10.2310/7290.2011.00001 21303611 PMC3629957

[B110] Tan Q. Zhao S. Xu T. Wang Q. Zhang M. Yan L. (2023). Inorganic nano-drug delivery systems for crossing the blood–brain barrier: advances and challenges. Coord. Chem. Rev. 494, 215344. 10.1016/j.ccr.2023.215344

[B111] Thangam R. Paulmurugan R. Kang H. (2021). Functionalized nanomaterials as tailored theranostic agents in brain imaging. Nanomaterials 12 (1), 18. 10.3390/nano12010018 35009968 PMC8746658

[B112] Toumia Y. Domenici F. Orlanducci S. Mura F. Grishenkov D. Trochet P. (2016). Graphene meets microbubbles: a superior contrast agent for photoacoustic imaging. ACS Appl. Mater. and interfaces 8 (25), 16465–16475. 10.1021/acsami.6b04184 27269868

[B113] Wang J. Zheng J. Yang Y. Liu X. Qiu J. Tian Y. (2022). Tunable full-color solid-state fluorescent carbon dots for light emitting diodes. Carbon 190, 22–31. 10.1016/j.carbon.2022.01.001

[B114] Wang W. Liu Z. Lan X. (2020). Quantum dot-based simultaneous multicolor imaging. Mol. Imaging Biol. 22, 820–831. 10.1007/s11307-019-01432-4 31529409

[B115] Winer J. L. Kim P. E. Law M. Liu C. Y. Apuzzo M. L. (2011). Visualizing the future: enhancing neuroimaging with nanotechnology. World Neurosurg. 75 (5-6), 626–637. 10.1016/j.wneu.2011.02.016 21704929

[B116] Wu D. Chen Q. Chen X. Han F. Chen Z. Wang Y. (2023). The blood–brain barrier: structure, regulation, and drug delivery. Signal Transduct. Target. Ther. 8 (1), 217. 10.1038/s41392-023-01481-w 37231000 PMC10212980

[B117] Yadollahpour A. L. I. Rashidi S. (2015). Magnetic nanoparticles: a review of chemical and physical characteristics important in medical applications. Orient J. Chem. 31 (1), 25–30. 10.13005/ojc/31.special-issue1.03

[B118] Yan L. Zhou X. Zheng Y. Luo W. Yang J. Zhou Y. (2019). Research progress in ultrasound use for the diagnosis and treatment of cerebrovascular diseases. Clinics 74, e715. 10.6061/clinics/2019/e715 30864640 PMC6438134

[B119] Yang H. Zhang C. Shi X. Hu H. Du X. Fang Y. (2010). Water-soluble superparamagnetic manganese ferrite nanoparticles for magnetic resonance imaging. Biomaterials 31 (13), 3667–3673. 10.1016/j.biomaterials.2010.01.055 20144480

[B120] Yang K. Hu L. Ma X. Ye S. Cheng L. Shi X. (2012). Multimodal imaging guided photothermal therapy using functionalized graphene nanosheets anchored with magnetic nanoparticles. Adv. Mater. 24 (14), 1868–1872. 10.1002/adma.201104964 22378564

[B121] Yang Y. Fan X. Li L. Yang Y. Nuernisha A. Xue D. (2020). Semiconducting polymer nanoparticles as theranostic system for near-infrared-II fluorescence imaging and photothermal therapy under safe laser fluence. Acs Nano 14 (2), 2509–2521. 10.1021/acsnano.0c00043 32022539

[B122] Yoon H. J. Lee E. S. Kang M. Jeong Y. Park J. H. (2015). *In vivo* multi-photon luminescence imaging of cerebral vasculature and blood–brain barrier integrity using gold nanoparticles. J. Mater. Chem. B 3 (15), 2935–2938. 10.1039/c4tb01759e 32262492

[B123] Yu F. Xu S. Ni X. Ye J. Cheng Y. Wang P. (2018) “Biomedical applications of functional micro-/nanoimaging probes,” in Advances in functional micro-/nanoimaging probes, 37–71.

[B124] Yu N. Zhao L. Cheng D. Ding M. Lyu Y. Zhao J. (2022). Radioactive organic semiconducting polymer nanoparticles for multimodal cancer theranostics. J. Colloid Interface Sci. 619, 219–228. 10.1016/j.jcis.2022.03.107 35397457

[B125] Zhang K. Xu H. Li K. (2023). Molecular imaging for early-stage disease diagnosis//visualized medicine: emerging techniques and develo** frontiers. Singapore: Springer Nature Singapore, 39–58.

[B126] Zhang P. Ma X. Guo R. Ye Z. Fu H. Fu N. (2021a). Organic nanoplatforms for iodinated contrast media in CT imaging. Molecules 26 (23), 7063. 10.3390/molecules26237063 34885645 PMC8658861

[B127] Zhang S. Zhou Y. Li R. Chen Z. Fan X. (2022). Advanced drug delivery system against ischemic stroke. J. Control. Release 344, 173–201. 10.1016/j.jconrel.2022.02.036 35248645

[B128] Zhang W. Mehta A. Tong Z. Esser L. Voelcker N. H. (2021b). Development of polymeric nanoparticles for blood–brain barrier transfer—strategies and challenges. Adv. Sci. 8 (10), 2003937. 10.1002/advs.202003937 PMC8132167 34026447

[B129] Zhang Y. García-Gabilondo M. Grayston A. Feiner I. V. J. Anton-Sales I. Loiola R. A. (2020). PLGA protein nanocarriers with tailor-made fluorescence/MRI/PET imaging modalities. Nanoscale 12 (8), 4988–5002. 10.1039/c9nr10620k 32057060

[B130] Zhang Y. Hong H. Myklejord D. V. Cai W. (2011). Molecular imaging with SERS‐active nanoparticles. Small 7 (23), 3261–3269. 10.1002/smll.201100597 21932216 PMC3228876

[B131] Zhang Y. Petibone D. Xu Y. Mahmood M. Karmakar A. Casciano D. (2014). Toxicity and efficacy of carbon nanotubes and graphene: the utility of carbon-based nanoparticles in nanomedicine. Drug metab. Rev. 46 (2), 232–246. 10.3109/03602532.2014.883406 24506522

[B132] Zhang Y. Wu M. Wu M. Zhu J. Zhang X. (2018). Multifunctional carbon-based nanomaterials: applications in biomolecular imaging and therapy. Acs Omega 3 (8), 9126–9145. 10.1021/acsomega.8b01071 31459047 PMC6644613

[B133] Zhao B. Ma H. Zheng M. Xu K. Zou C. Qu S. (2022b). Narrow‐bandwidth emissive carbon dots: a rising star in the fluorescent material family. Carbon Energy 4 (1), 88–114. 10.1002/cey2.175

[B134] Zhao W. Yu X. Peng S. Luo Y. Li J. Lu L. (2021). Construction of nanomaterials as contrast agents or probes for glioma imaging. J. Nanobiotechnology 19 (1), 125. 10.1186/s12951-021-00866-9 33941206 PMC8091158

[B135] Zhao X. Bian F. Sun L. Cai L. Li L. Zhao Y. (2020). Microfluidic generation of nanomaterials for biomedical applications. Small 16 (9), 1901943. 10.1002/smll.201901943 31259464

[B136] Zhao Z. Li M. Zeng J. Huo L. Liu K. Wei R. (2022a). Recent advances in engineering iron oxide nanoparticles for effective magnetic resonance imaging. Bioact. Mater. 12, 214–245. 10.1016/j.bioactmat.2021.10.014 35310380 PMC8897217

[B137] Zheng J. Cheng X. Zhang H. Bai X. Ai R. Shao L. (2021). Gold nanorods: the most versatile plasmonic nanoparticles. Chem. Rev. 121 (21), 13342–13453. 10.1021/acs.chemrev.1c00422 34569789

[B138] Zielińska A. Carreiró F. Oliveira A. M. Neves A. Pires B. Venkatesh D. N. (2020). Polymeric nanoparticles: production, characterization, toxicology and ecotoxicology. Molecules 25 (16), 3731. 10.3390/molecules25163731 32824172 PMC7464532

